# The *Aspergillus nidulans* MAPK Module AnSte11-Ste50-Ste7-Fus3 Controls Development and Secondary Metabolism

**DOI:** 10.1371/journal.pgen.1002816

**Published:** 2012-07-19

**Authors:** Özgür Bayram, Özlem Sarikaya Bayram, Yasar Luqman Ahmed, Jun-ichi Maruyama, Oliver Valerius, Silvio O. Rizzoli, Ralf Ficner, Stefan Irniger, Gerhard H. Braus

**Affiliations:** 1 Institute of Microbiology and Genetics, Department of Molecular Microbiology and Genetics, Georg-August-Universität, Göttingen, Germany; 2 Department of Molecular Structural Biology, Institute for Microbiology and Genetics, Georg-August-Universität, Göttingen, Germany; 3 European Neuroscience Institute, Deutsche Forschungsgemeinschaft Center for Molecular Physiology of the Brain/Excellence Cluster 171, Göttingen, Germany; University of California San Francisco, United States of America

## Abstract

The sexual Fus3 MAP kinase module of yeast is highly conserved in eukaryotes and transmits external signals from the plasma membrane to the nucleus. We show here that the module of the filamentous fungus *Aspergillus nidulans* (An) consists of the AnFus3 MAP kinase, the upstream kinases AnSte7 and AnSte11, and the AnSte50 adaptor. The fungal MAPK module controls the coordination of fungal development and secondary metabolite production. It lacks the membrane docking yeast Ste5 scaffold homolog; but, similar to yeast, the entire MAPK module's proteins interact with each other at the plasma membrane. AnFus3 is the only subunit with the potential to enter the nucleus from the nuclear envelope. AnFus3 interacts with the conserved nuclear transcription factor AnSte12 to initiate sexual development and phosphorylates VeA, which is a major regulatory protein required for sexual development and coordinated secondary metabolite production. Our data suggest that not only Fus3, but even the entire MAPK module complex of four physically interacting proteins, can migrate from plasma membrane to nuclear envelope.

## Author Summary

Mitogen activated protein (MAP) kinase cascades are conserved from yeast to man to transmit an external signal to the nucleus and induce an appropriate cellular response. The yeast Fus3 MAP kinase module represents a textbook paradigm for signal transduction. The pathway is activated by external sexual hormones triggering several kinases that transmit the signal at the plasma membrane to Fus3. Phosphorylated Fus3 is released from the membrane-associated module, crosses the cytoplasm, and enters the nucleus to activate transcription factors for sexual development. We describe here the Fus3 MAPK pathway of a filamentous fungus that controls sexual development as well as secondary metabolism, which are coordinated processes in filamentous fungi. *Aspergillus nidulans* is able to release Fus3 as a complex from the membrane. Complexes of Fus3 can include two additional kinases and an adaptor protein, and these complexes can migrate from the membrane to the nuclear envelope where only *A. nidulans* Fus3 can enter the nucleus to control nuclear regulators. Revealing specific functions of cellular Aspergillus Fus3 complexes in signal transduction to control fungal development and secondary metabolism will be a fascinating future task.

## Introduction

Eukaryotic organisms communicate between cell surface and nucleus to respond to environmental signals. The mitogen-activated protein kinase (MAPK) module consisting of a cascade of three protein kinases represents a highly conserved eukaryotic signal transduction system present from yeast to man. MAP3K phosphorylates a second kinase, MAP2K which itself phosphorylates the MAPK. This final kinase phosphorylates nuclear target proteins to activate appropriate gene expression [1], [2].

The sexual pathway of the budding yeast *Saccharomyces cerevisiae* represents a paradigm for signal transduction in eukaryotes [3]–[5]. This MAP kinase pathway responds to pheromones and induces differentiation processes which trigger sexual mating of yeast [4], [6]. The central complex of MAP3K Ste11, MAP2K Ste7 and MAPK Fus3 is assembled on the scaffold protein Ste5 as a hub to keep these kinases in a close proximity for enhanced relay of phosphorylation and thereby controls the flow of information [7]. Binding of pheromone to the transmembrane receptors Ste2 or Ste3, which are coupled to guanine nucleotide binding proteins (G protein, G protein coupled receptor: GPCR), initiates signal transduction. This induces the release of the Gβγ subunit from the trimeric Gαβγ protein. The Ste5 RING domain binds to activated free Gβγ complex and recruits the MAP kinase module Ste11-Ste7-Fus3 to the membrane [8]–[10] in close distance to the p21 activated kinase (PAK) Ste20. Preactivated Ste20 is localized in the membrane and initiates the kinase cascade system by phosphorylating the MAP3K Ste11 [4].

Ste50 represents a second adaptor which binds to the Opy2 membrane anchor and provides membrane association of the entire MAPK module. Ste50 mediated membrane localization is required for Ste11 activation [11], [12]. The information is transmitted as phosphate signal from Ste11 via Ste7 to the MAPK Fus3. According to the current model phosphorylated Fus3 is released from the Ste5 scaffold complex and leaves the membrane associated complex [13]–[15]. Phosphorylated Fus3 crosses the cytoplasm and enters the nucleus where it phosphorylates target transcription factors as Ste12. Ste12 is necessary to activate the sexual pathway and also controls developmental processes [4], [5].

**Figure 1:**
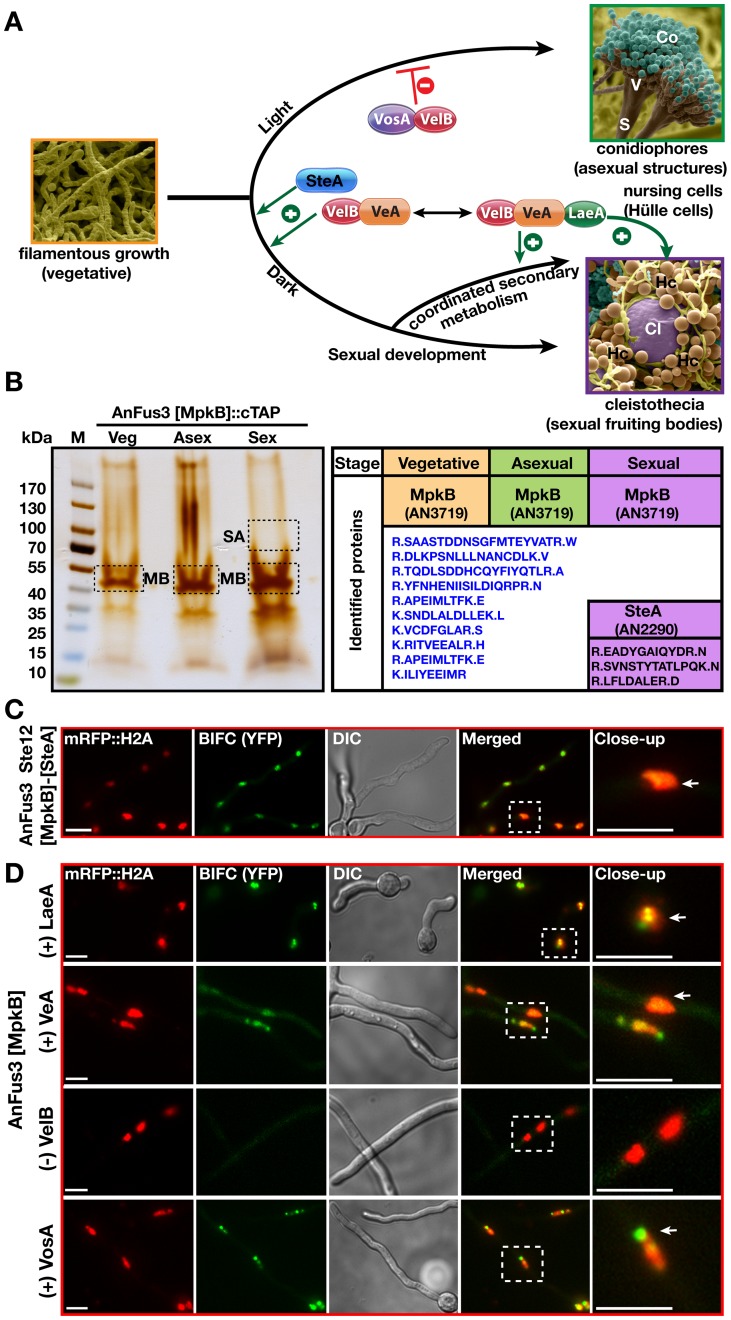
Identification of AnFus3 [MpkB] associated proteins and AnFus3 interactions with the velvet complex components. (A) Life cycle of *Aspergillus nidulans* and developmental functions of AnSte12 [SteA], LaeA and velvet domain proteins. Germination of spores leads to tube-like vegetative filaments (hyphae) which become competent for environmental signals after at least 12 hours of growth. Exposure of developmentally competent hyphae to light (or aeration) leads to asexual development (conidiophores and asexual spores [conidia]) in 24 hours. VosA-VelB inhibits asexual differentiation. Incubation in dark (96 hours) induces the sexual cycle with sexual fruiting bodies (cleistothecia) which are nursed by globose Hülle cells. LaeA is required for Hülle cell formation. VelB-VeA supports sexual development together with AnSte12 [SteA]. The VelB-VeA-LaeA trimeric complex coordinates development with secondary metabolism. Co; conidia, S; stalk, Cl; cleistothecium, Hc; Hülle cells. (B) A silver stain treated 5–14% gradient SDS polyacrylamide gel of AnFus3 [MpkB]::cTAP from vegetatively, asexually (on plates, under light) and sexually (on plates, in the dark) grown cultures at 30°C for 20 hours. Identified proteins from the excised lanes (Table S1). SA; SteA-AnSte12, MB; MpkB-AnFus3. (C) AnFus3-AnSte12 interaction *in vivo*. N-EYFP::AnFus3 [MpkB] fusion interacts with C-EYFP fusion of AnSte12 [SteA] in the nuclei (arrow) which were visualized by a monomeric red fluorescent protein histone 2A fusion (mRFP::Histone2A). (D) Interaction partners of AnFus3 [MpkB] in BIFC. (+) indicates AnFus3 interactions with VosA and LaeA at very early stages after germination (10–12 hours) and with VeA after 24 hours of hyphal growth. (−) indicates that VelB does not interact with AnFus3. Scale bars are 10 µm.

Pheromone pathway genes have been studied in various fungi and are not only involved in sexual reproduction but also in fungal pathogenicity [16]–[21]. The Fus3 MAPK module is highly conserved in filamentous fungi with the exception that homologs for Ste5 are absent [22], [23]. In the self-fertile model fungus *Aspergillus nidulans*, the Ste11 MAP3K homolog SteC (AnSte11) [24], the Fus3 MAPK homolog MpkB (AnFus3) [25], and the Ste12 homolog for the transcription factor SteA (AnSte12) [26] are necessary for sexual fruiting body formation, suggesting that there are similarities in the molecular function of the MAPK signal transduction as in yeast. *A. nidulans* grows vegetatively as a filament. When placed on a surface, after germination of the spores at least 12 hours of growth is required to establish developmental competence in response to external signals [27]. There are two developmental options: light supports the asexual and inhibits the sexual developmental pathway (Figure 1A). AnFus3 is not only required for sexual development but also for the control of secondary metabolism which is a typical feature of many filamentous fungi [28]. Sexual development of *A. nidulans* is coordinated with the production of secondary metabolites, including mycotoxins. This coordination requires velvet domain proteins which are common for filamentous fungi but absent in yeast [6]. The velvet heterodimers VeA-VelB and VosA-VelB have different developmental functions. VeA-VelB heterodimer promotes sexual development whereas VelB-VosA dimer inhibits asexual differentiation. Association of the putative methyltransferase LaeA [29] with the VelB-VeA heterodimer, which makes the VelB-VeA-LaeA trimeric complex, coordinates development and secondary metabolism [6], [30], [31].

Comparison of the intracellular molecular mechanism of signal transduction of Fus3 MAPK of yeast and *A. nidulans* revealed that AnFus3 MAPK can reach the nuclear envelope in a complex with other proteins of the MAPK module, including the adaptor protein AnSte50. Only AnFus3 enters the nucleus and phosphorylates VeA, which elucidates a novel link between MAPK and velvet domain proteins that act as control elements at the interface of fungal development and secondary metabolism.

## Results

### The *A. nidulans* Fus3 MAP kinase of the mating pathway phosphorylates the velvet domain protein VeA, and VelB-VeA complex formation is reduced in *Anfus3* deletion

*S. cerevisiae* Fus3 interacts with transcription factor Ste12 that activates the mating pathway. The *A. nidulans* MAP kinase AnFus3 [MpkB] also controls sexual development [25], [28], [32]. Tagged AnFus3 recruited the transcription factor AnSte12 [SteA] by tandem affinity purification (TAP) only when the fungus was induced for sexual development but not during vegetative filamentous growth or asexual development (Figure 1B, Table S1). Endogenously expressed AnFus3::sGFP was functional (Figure S1) and immunoprecipitation of the fusion protein was able to enrich the SteA protein in a sexually induced culture (Table S1). The AnFus3-SteA interaction was further verified by bimolecular fluorescence complementation (BiFC) and was observed in fungal nuclei (Figure 1C). This corroborates that the interaction between kinase and transcription factor is conserved from yeast to filamentous fungi.

Due to their similar roles in development and secondary metabolism [28]–[30], we examined whether AnFus3 interacts with the velvet domain proteins and LaeA. AnFus3 interacted *in vivo* in a BiFC assay with LaeA and subsequently with VeA, but not with VelB. In addition, AnFus3 interacted with VosA (Figure 1D). VosA is part of the VosA-VelB heterodimer which represses asexual development [31], [33]. These results suggest that distinct velvet domain proteins or LaeA may include targets of MAPK phosporylation.

**Figure 2:**
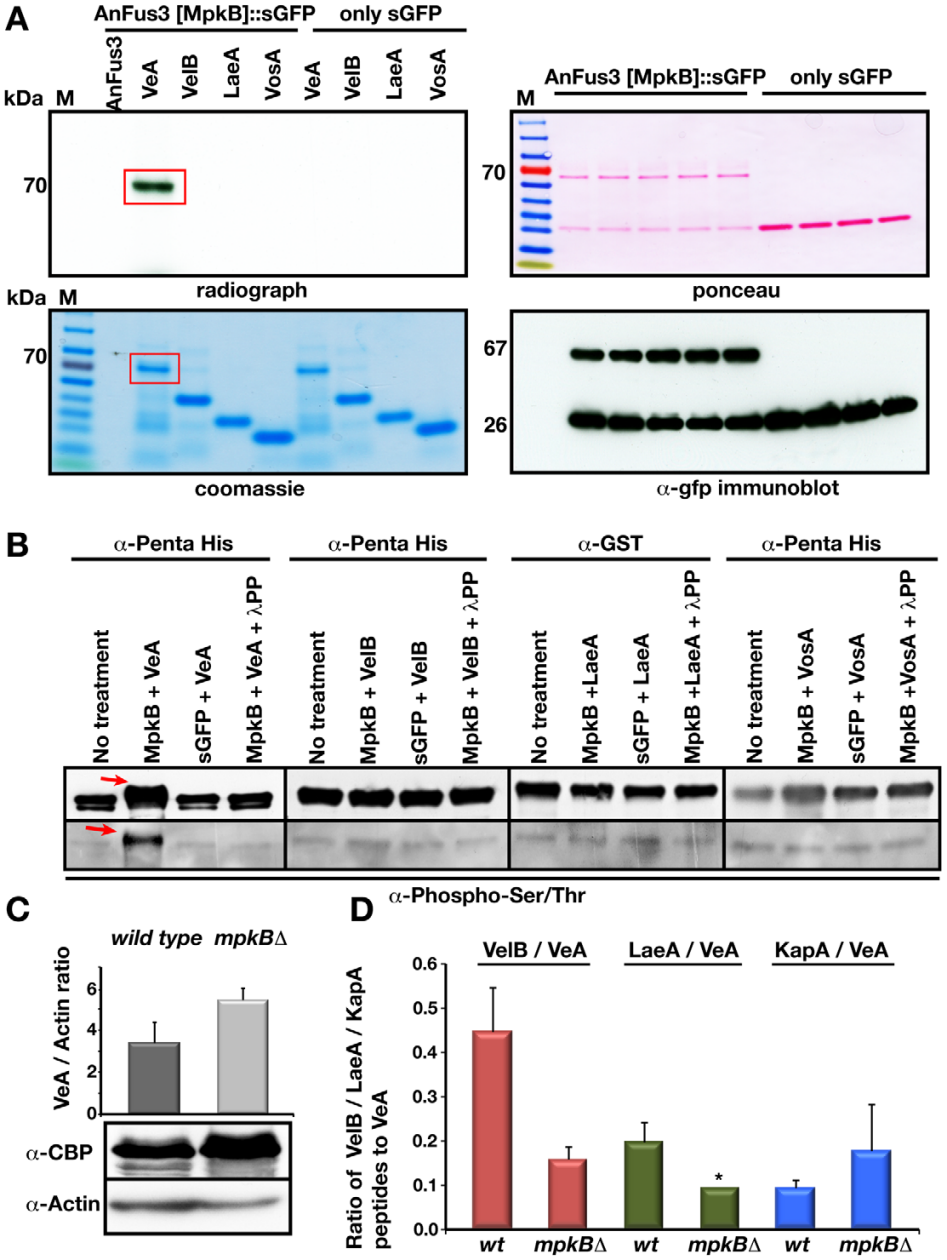
Phosphorylation of VeA by the MAP kinase AnFus3 [MpkB] and influence of AnFus3 on the interactions of the velvet complex. (A) *In vitro* phosphorylation of VeA by AnFus3 in a radioactive kinase assay. Left panels; autoradiograph of dried SDS gel run for phosphorylation reactions (30 µl of total 45 µl reaction volume), Coomassie stain of the proteins from phosphorylation reaction (10 µl of total 45 µl reaction). VeA protein in AnFus3 reaction tube is shown with red rectangle. Right panels; ponceau staining of the immunoprecipitated (immobilized to the GFP trap sepharose) AnFus3::sGFP and only sGFP protein. Immunodetection of the fusion protein and free sGFP by the α-gfp. (B) Confirmation of specific VeA phosphorylation by a non-radioactive method. All recombinant proteins (10 µg each) were treated with both AnFus3 and GFP. AnFus3 treated samples were additionaly incubated with the lambda protein phosphatase (λ-PP). Proteins were immunodetected by α-Penta His, α-GST. After AnFus3 treatment, VeA showed a 3–5 kDA molecular weight shift (red arrow) that disappeared after L-PP treatment. Only VeA treated with MAPK was recognized by P-ser/thr specific antibody. (C) Protein levels of VeA in the wild type and *mpkB* mutant background. VeA::cTAP signals were normalized to the internal actin levels. VeA protein levels did not change in the absence of MpkB. (D) Reduced velvet complex formation in the *mpkB* mutant. The VeA-associated proteins from the cultures of the wild type and *mpkB*Δ strains grown in the darkness sexually at 30°C for 20 hours. Three independent experiments were performed and the associated proteins were identified. The ratio of the peptides from VelB and LaeA to the VeA protein drastically reduced in the MAPK mutant, whereas alpha importin KapA interaction slightly increased. Black bars represent the standard error. *LaeA was only found in one of the three purifications in *mpkB*Δ strain, thus no error bar is assigned.

AnFus3 was immunoprecipitated from vegetatively grown fungal cells as sGFP fusion protein (Figure 2A) to identify direct substrates of AnFus3 in *in vitro* kinase assays. VeA expressed and purified from *E. coli* was the only tested protein which could be specifically phosphorylated by AnFus3, whereas bacterially produced VosA, LaeA or VelB were not phosphorylated. Further phosphorylation experiments performed with phospho-specific serine and threonine antibodies further supported that VeA was phosphorylated by AnFus3 and treatment of phosphorylated samples with lambda protein phosphatase (λ-PP) resulted in loss of phosphorylation signal (Figure 2B).

VeA bridges VelB and LaeA in the trimeric VelB-VeA-LaeA complex. We addressed whether AnFus3 activity affects complex formation. VeA protein levels (Figure 2C) were similar in wild type and *mpkB* mutant strains. *velB* RNA was unchanged whereas *laeA* transcripts were downregulated as previously reported (Figure S2A) [28]. TAP purification of natively expressed VeA::cTAP revealed that under conditions where sexual development was normally promoted, only significantly reduced amounts of VelB and LaeA proteins were enriched by tagged VeA in the absence of MpkB (Figure 2D, Tables S2 and S3). The MAP kinase does not affect VeA nuclear import, because the interaction of VeA with the importin KapA was not significantly affected in *mpkB* mutant. Consistently, nuclear import of the subunits of the trimeric VelB-VeA-LaeA complex was not affected in a *mkkB* mutant lacking the upstream MAP2K AnSte7 (Figure S2B). Lack of *laeA* normally causes enhanced VeA and VelB expression as well as enhanced complex formation [31]. This suggests that decreased VeA-VelB association is not a result of the reduced levels of LaeA in *mpkB* mutants.

These results suggest that AnFus3 phosphorylates VeA *in vitro* and interacts with VeA *in vivo*. Furthermore, AnFus3 is required for enhanced association of VeA with VelB which are components of the VelB-VeA-LaeA velvet complex.

### MAP2K AnSte7 is required for sexual development of *A. nidulans*

**Figure 3:**
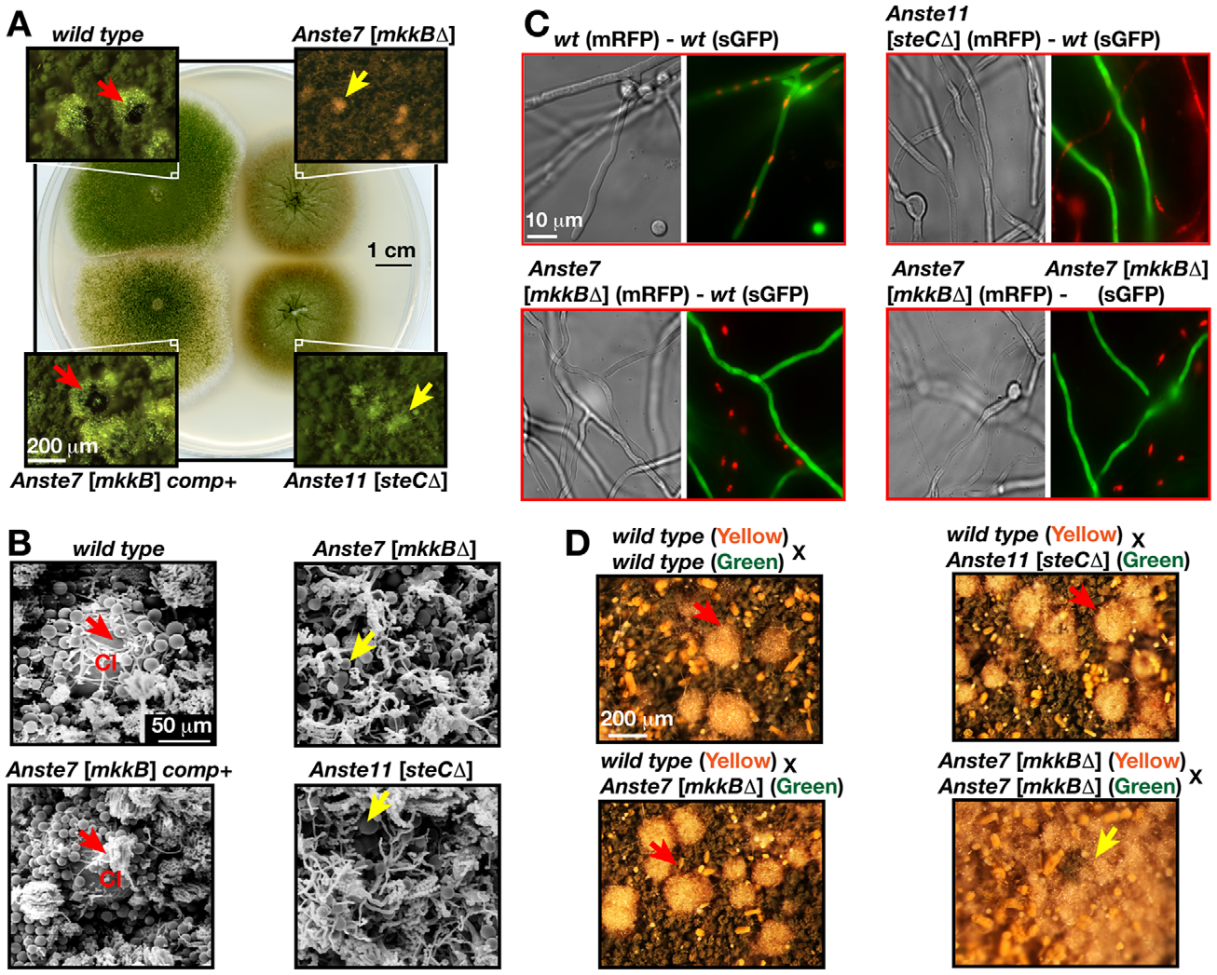
Loss of sexual fruiting bodies and heterokaryon formation in *mkkB*Δ strain lacking AnSte7. (A) Sexual developments of a wild type, *mkkB*Δ, *mkkB* complementation (comp+), *steC*Δ [lacking AnSte11] strains, which were point inoculated (1×10^4^) and grown on minimal medium in dark conditions (5 days at 37°C). Small squares are the close-up stereomicroscopic images of the strains. Red arrows indicate the mature black fruiting bodies of the wild type and complementation strains and yellow arrows denote the premature nests produced by *mkkB* **Δ** and *steC* **Δ** strains. (B) Raster electron microscopy (REM) image of the strains from (A). The wild type fruiting body (cleistothecium: Cl) is surrounded by the globose Hülle cells. *steC* and *mkkB* mutants produce only dispersed groups of Hülle cells (yellow arrows) instead of mature fruiting bodies. (C) Monitoring hyphal fusions and heterokaryons via fluorescence microscopy. Strains bearing either cytoplasmic synthetic green fluorescent protein (sGFP) or nuclear red fluorescent protein fused histone 2A (mRFP) were used in different combinations. Only two wild types form green and red fluorescent combinations through hyphal fusions. (D) Isolated protoplasts from two wild types (yellow and green), *steC*Δ (green), *mkkB*Δ (yellow or green) were used for protoplast fusions as shown in combinations and plated on selective medium. *wt*/*wt*, *wt*/*steC*Δ, *wt*/*mkkB*Δ combinations produce fruiting bodies after 7–8 days, whereas *mkkB* **Δ**/*mkkB* **Δ** combination only produces nests.

MAPKKK (SteC) and MAPK (MpkB) are necessary for sexual development in *A. nidulans* [24], [25]. Yeast Fus3 receives the phosphorylation signal from MAP2K Ste7. The corresponding filamentous fungus homolog has not yet been described. The ANID_03422 (*mkkB*) locus of *A. nidulans* encodes a protein, which is conserved in different Aspergilli (Figure S3) and has 25% identity to yeast Ste7 [34]. AnSte7 [MkkB] is also related to *N. crassa* MAP2K [35] and human MAP2K1 [36]. Overexpressing the corresponding *mkkB* gene resulted in two fold increase in the number of fruiting bodies and supported a role in sexual development (Figure S4A–S4C). *mkkB* deletion mutants had a slow growth phenotype and were blocked in early sexual development, which resulted in nest-like structures containing clumps of Hülle cells (yellow arrows, Figure 3A, 3B). Hülle cells support sexual development as specialized nursing cells for the growing fruiting body [31].

AnSte7 is required for hyphal fusion as one of the initial steps of fruiting body formation. Hyphal fusion of wild type strains marked with either synthetic cytoplasmic green fluorescent protein (sGFP) or with nuclear monomeric red fluorescent protein (mRFP) resulted in hyphae with green cytoplasm and red nuclei (heterokaryon) (Figure 3C). In contrast, a *mkkB* deletion strain was unable to fuse with the wild type strain. We found the same hyphal fusion defect for the *steCΔ* strain as in the *mkkB* mutant (Figure 3C, 3D). This further supports that AnSte11 and AnSte7 act in a common pathway.

The analysis of putative additional functions of AnSte7 in later phases of sexual development required a by-pass of initial hyphal fusions. Therefore, heterokaryons were artificially produced by fusing protoplasts. An intact *mkkB* copy of the wild type strain allowed the development of mature fruiting bodies (red arrows), when wild type and *mkkB* mutant protoplasts were fused. In contrast, two *mkkB* mutants forced to form heterokaryons were impaired in fruiting body maturation and produced only early structures of development (yellow arrow, Figure 3D). This suggests several functions of MAP2K AnSte7 during sexual development presumably in concert with AnFus3.

### AnSte50-Ste11-Ste7-Fus3 form a physically interacting module that is required for sexual development

We determined whether the *A. nidulans* kinases may replace functions of its yeast counterparts. Plasmids containing *Anste7* [*mkkB*] and *Anfus3* [*mpkB*] genes expressed under yeast promoters were transformed into *ste7* and *fus3* deletion strains. *mkkB* and *mpkB* did not alleviate the defects in pheromone response of the yeast mutants (Figure S4D). However, MpkB moderately suppressed the defects in pheromone response of a *fus3 kss1* double mutant, showing that the MpkB is partially able to take over functions of the MAP kinase pair Fus3/Kss1. This suggests a partial overlap of the functions of the MAPK pathways of these two organisms.

**Figure 4:**
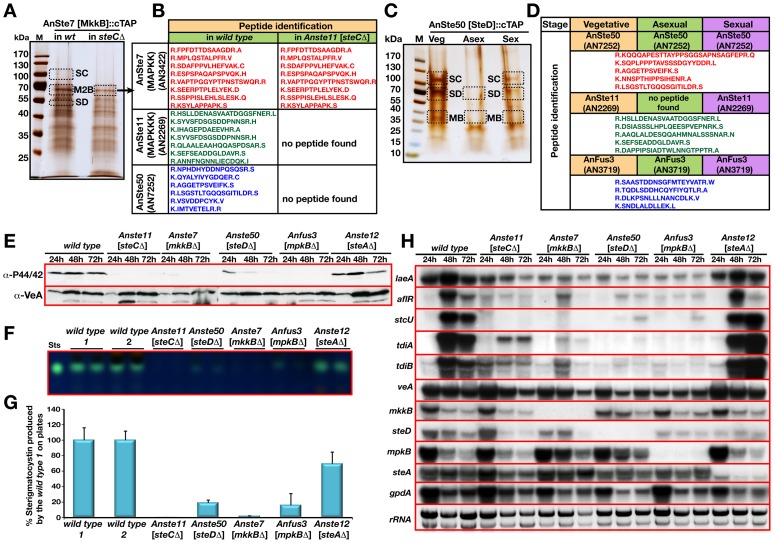
Identification of AnSte7 [MkkB], and AnSte50 [SteD] associated proteins and role of MAPK pathway on secondary metabolism. (A) A silver stained 5–14% gradient SDS polyacrylamide gel of AnSte7 [MkkB]::cTAP from the wild type and *steC*Δ [lacking AnSte11] background grown for vegetatively at 30°C for 20 hours. (B) Identified peptides of the proteins from the excised lanes of the wild type and *steC*Δ strains (Table S4 and S5). AnSte11 [SteC] (AN2269) and AnSte50 [SteD] (AN7252) were found as interactors of AnSte7 [MkkB]. (C) Interaction partners of the AnSte50::cTAP fusion from vegetatively, asexually and sexually grown cultures at 30°C for 20 hours (Table S6) (M2B; MkkB, MB; MpkB, SD; SteD, SC; SteC). (D) Identified polypeptides from the bands. (E) Monitoring of the phosphorylation status of the MpkB by phospho-p44/42 MAPK (Thr182/Tyr184) antibody in the wild type as well as pheromone pathway mutants grown for 24, 48 and 72 hours vegetatively. VeA protein levels served as loading control. 80 µg protein extract was loaded on each lane. (F) Production of secondary metabolite sterigmatocystin (ST) in the mutants of pheromone pathway, *Anste11* [*steC*Δ], *Anste50* [*steD*Δ], *Anste7* [*mkkB*Δ], *Anfus3* [*mpkB*Δ], *Anste12* [*steA*Δ], respectively. Developed TLC plates show sterigmatocystin production. Sts; Sterigmatocystin standard. (G) Quantification of the ST production from the TLC plates. Wild type ST levels served as 100% standard. (H) Expression of the developmental, secondary metabolite genes in the pheromone pathway mutants. *laeA, aflR, stcU* for ST production, *tdiA* and *tdiB* for terrequinone (TQ) production and *veA, mkkB, steD, mpkB, steA* for developmental purposes were monitored. Strains were grown in the liquid medium for 24, 48 and 72 hours and total RNA was isolated and blotted (20 µg). Glycolytic gene *gpdA* expression and ethidium bromide stained rRNA was used as loading control.

The *A. nidulans* MAP kinase mating module was further characterized by identifying interaction partners of AnSte7 [MkkB] by TAP purification from different developmental stages (only vegetative is shown, Figure 4A, 4B, Figure S5, Tables S4, S5). Tagged AnSte7 did not recruit AnFus3, but copurified AnSte11 [SteC] and AnSte50 [SteD], a protein sharing homology to *S. cerevisiae* Ste50.

Ste50 functions as an adaptor for membrane recruitment of Ste11 in yeast [12]. Deletion of the corresponding *steD* in *A. nidulans* caused a defect in fruiting body formation (Figure S1A). Similar to the other MAPK mutants, *steD* mutant could not produce heterokaryons in outcrossings (not shown). Thus, the adaptor AnSte50 is as important for accurate fungal development as the other components of the MAPK module. *A. nidulans* AnSte50 was enriched by AnSte7::TAP in wild type, but not in the *steCΔ* strain indicating that AnSte11 is required for the AnSte50-Ste7 interaction (Figure 4A, 4B). These data suggest a physical interaction of AnSte50 and two MAPK module components in a AnSte50-Ste11-Ste7 complex.

Interaction partners of AnSte50 were identified to explore the entire fungal MAPK mating module. A functional *steD::ctap* (Figure S1A, S1B) recruited the MAP3K AnSte11 and the MAPK AnFus3 but not the MAP2K AnSte7 (Figure 4C, 4D and Table S6). This further supports that AnSte50-Ste11-Ste7-Fus3 forms a module similar to yeast Ste5-Ste50-Ste11-Ste7-Fus3 with the exception that a counterpart for the yeast Ste5 scaffold is missing in *A. nidulans*.

### AnSte50-Ste11-Ste7-Fus3 represents an active MAP kinase module required for sexual development and secondary metabolite synthesis

We analysed whether AnSte11 and AnSte7 act upstream of MAPK AnFus3. MAPK phosphorylation was monitored by a phospho-specific antibody against the MAPK Thr182XTyr184 motif. Phosphorylated AnFus3 was permanently detectable in vegetative wild type cultures (Figure 4E). In contrast, modified AnFus3 was absent in mutants lacking AnSte11 or AnSte7, whereas the absence of AnSte12 did not change levels of phosphorylated AnFus3. In the absence of AnSte50, reduced phosphorylation of AnFus3 indicates some residual activity of the untethered AnSte11-Ste7 complex. This supports an active *A. nidulans* MAPK module consisting of AnSte50-Ste11-Ste7-Fus3 which controls fungal sexual development.

The role of AnSte50-Ste11-Ste7-Fus3 for secondary metabolism was examined. Impaired secondary metabolism had only been described for the *mpkB* mutants [28]. The mycotoxin sterigmatocystin (ST) levels were drastically reduced in the sterile *steC*, *steD, mkkB*, or *mpkB* mutants whereas ST levels in the sterile *steA*Δ [AnSte12] were similar to wild type (Figure 4F–4G). Similarly, the expression of the biosynthesis genes for ST (*stcU*) and terrequinone (*tdiA* and *tdiB*), and the expression of *laeA* and the transcription factor encoding *aflR*, both required for expression of secondary metabolite genes, were distinctly reduced in each mutant of the MAPK module (Figure 4H). These data corroborate that active AnSte50-Ste11-Ste7-Fus3 MAPK is not only required for sexual development but also for secondary metabolite production.

### The components of the fungal MAPK module exhibit distinct localization patterns at hyphal tip, nuclear envelope, and septa

The yeast mating MAPK module transmits a signal from the plasma membrane to the nucleus by releasing MAPK Fus3 from the Ste5 scaffold at the membrane [13], [37]. We analysed how the signal is transmitted through the filament of *A. nidulans* to nuclear factors as AnSte12 or VeA. Time course immunoblotting (Figure S1C) showed that AnFus3 was constantly expressed during development. The *mkkB* mRNA for the upstream MAPKK was also present throughout all stages (Figure S5C). The corresponding protein AnSte7::sGFP was present in vegetative as well as in the initial phases of asexual and sexual development, but decreased afterwards (Figure S5C). Similarly, the AnSte50::sGFP (Figure S1C) seems to be degraded because the protein disappeared during mid and late asexual development.

**Figure 5:**
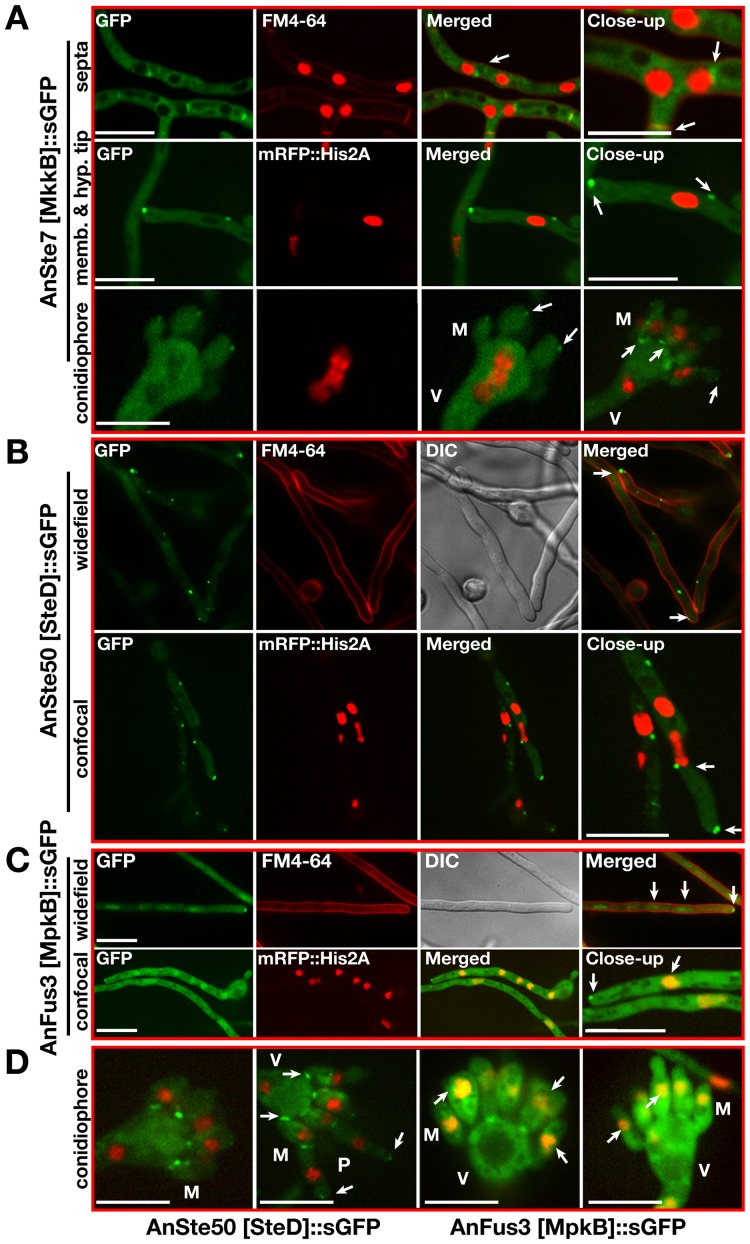
Subcellular localizations of AnSte7 [MkkB], AnSte50 [SteD], and AnFus3 [MpkB]::sGFP fusions during the development of the fungus. (A) Subcellular location of the AnSte7 [MkkB]::sGFP fusion protein in vegetatively and asexually growing hyphae. mRFP::Histone2A fusion indicates the position of the nuclei and FM4-64 stains the plasma membrane and the septa of the hyphae. Arrows indicate the accumulation of the fusion protein at hyphal tip, membrane and septa of the vegetative hyphae and metulae (M) of the conidiophores. (V) indicates the swollen vesicle part of the conidiophore. (B) Localization of AnSte50 [SteD]::sGFP fusion in the hyphal cells. SteD protein is cytoplasmic and after competence time (16 hours) it is enriched at the hyphal tip, membrane and nuclear envelope (indicated by arrows). (C) Nucleo-cytoplasmic and hyphal tip distribution of endogenously expressed AnFus3 [MpkB]::sGFP kinase fusion protein in the fungal hyphae. AnFus3 is found in the cytoplasm and nucleus, in late hours of vegetative growth (after 16 hours) accumulates at the plasma membrane and hyphal tips. (D) Presence of SteD protein at the base of the metulae and in the septa between the metulae (M) and phialides (P). Nuclear and partial septal localization of the AnFus3 protein in the asexual structures (arrows). Scale bars represent 10 µm.

Confocal spinning disc microscopy revealed that functional AnSte7::sGFP fusion protein expressed under native locus promoter was localised during early phase of growth throughout the cytoplasm, but never found in the nucleus (not shown). After becoming competent for differentiation (16 hours after germination), AnSte7::sGFP accumulated not only at the hyphal tip but also at the plasma membrane and at the septa of hyphae or spore forming cells (white arrows in Figure 5A). The AnSte7 signal was also present on the nuclear envelope. The AnSte7 localization pattern did not change in the absence of the MAP3K AnSte11 (not shown). Like AnSte7, a functional Ste50::sGFP fusion never entered the nucleus. AnSte50 was cytoplasmic and accumulated at later stages of vegetative growth at the hyphal tip, the septa of spore forming cells, the plasma membrane and the nuclear envelope (arrows in Figure 5B, 5D).

A functional AnFus3::sGFP expressed under the native promoter accumulated at the hyphal tip and was as well present in the cytoplasm as in the nucleus in vegetative and spore forming cells (Figure 5C, 5D). This suggests a dynamic and complex distribution of MAPK module subunits from the fungal membrane to the nucleus. It also revealed that the MAPK AnFus3 is as yeast Fus3 the only subunit with the potential to enter the nucleus.

### The entire MAPK module colocalizes and interacts at hyphal tip and nuclear envelope

**Figure 6:**
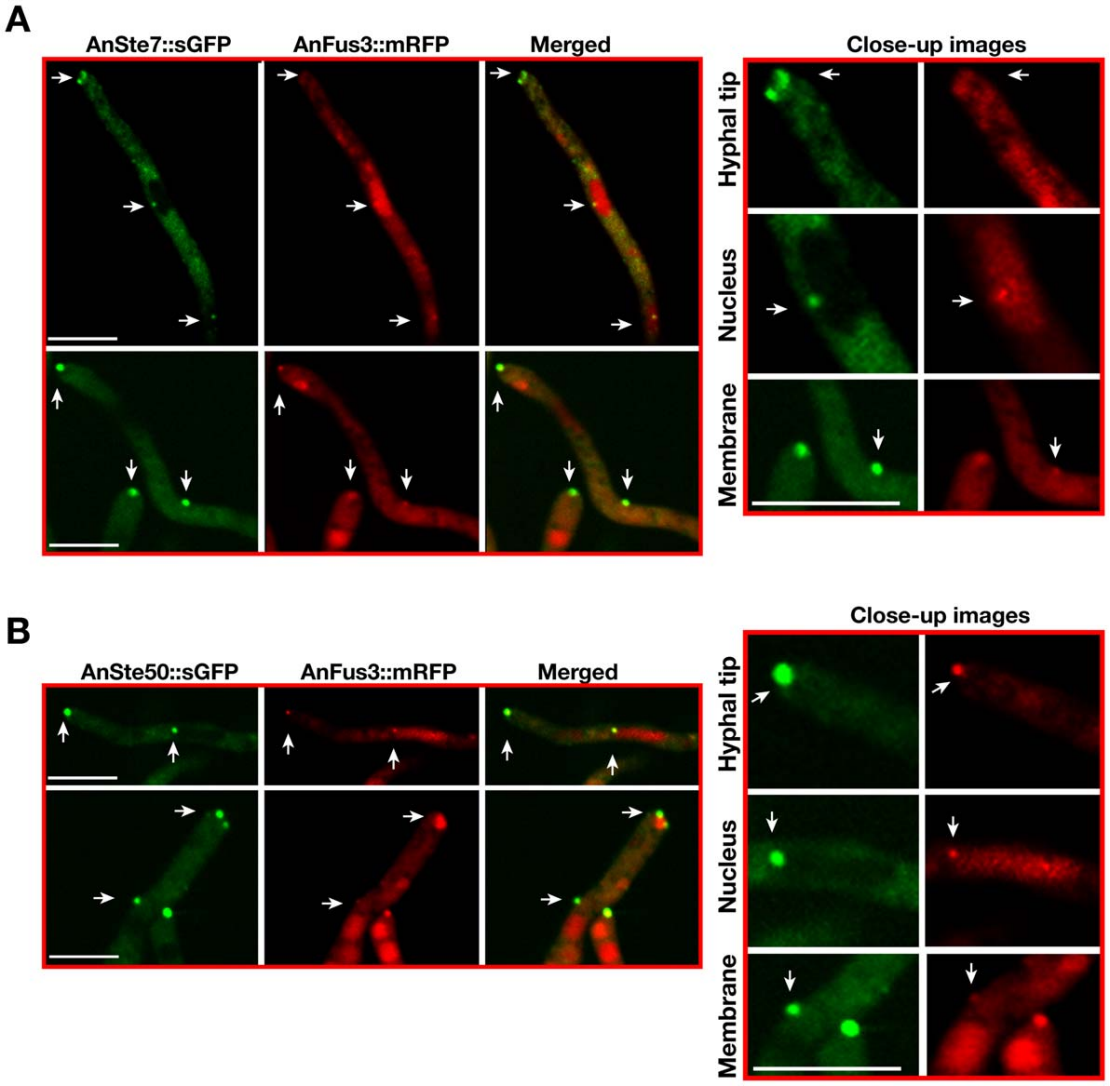
Colocalizations of AnSte7 or AnSte50::sGFP with AnFus3::mRFP. (A) Colocalizations of AnSte7 and AnFus3 proteins within the same fungal cell. White arrows indicate AnSte7::sGFP and AnFus3::mRFP colocalizations at the hyphal tip, membrane and on the nuclear envelope. (B) Colocalizations of AnSte50 and AnFus3 proteins within the same cell at the hyphal tip, plasma membrane and perinuclear district. Scale bars are 10 µm.

AnFus3 [MpkB]::mRFP was expressed constitutively together with AnSte7 [MkkB] and AnSte50 [SteD]::sGFP fusions to validate whether all components of the MAPK module are colocalized within the fungal filament (Figure 6). Most of the GFP signals of AnSte7 and Ste50 merge with the RFP signal of MpkB at the fungal tip, the plasma membrane and at the nuclear envelope where they might form dynamic protein complexes. Exclusively at hyphal tips we found two types of co-localizations of kinase pairs. In addition to direct co-localizations, similar to plasma membrane or nuclear envelope, there were extended co-localization patterns at the hyphal tip. This could reflect that a fraction of kinases is localized in vesicles at the hyphal tip.

**Figure 7:**
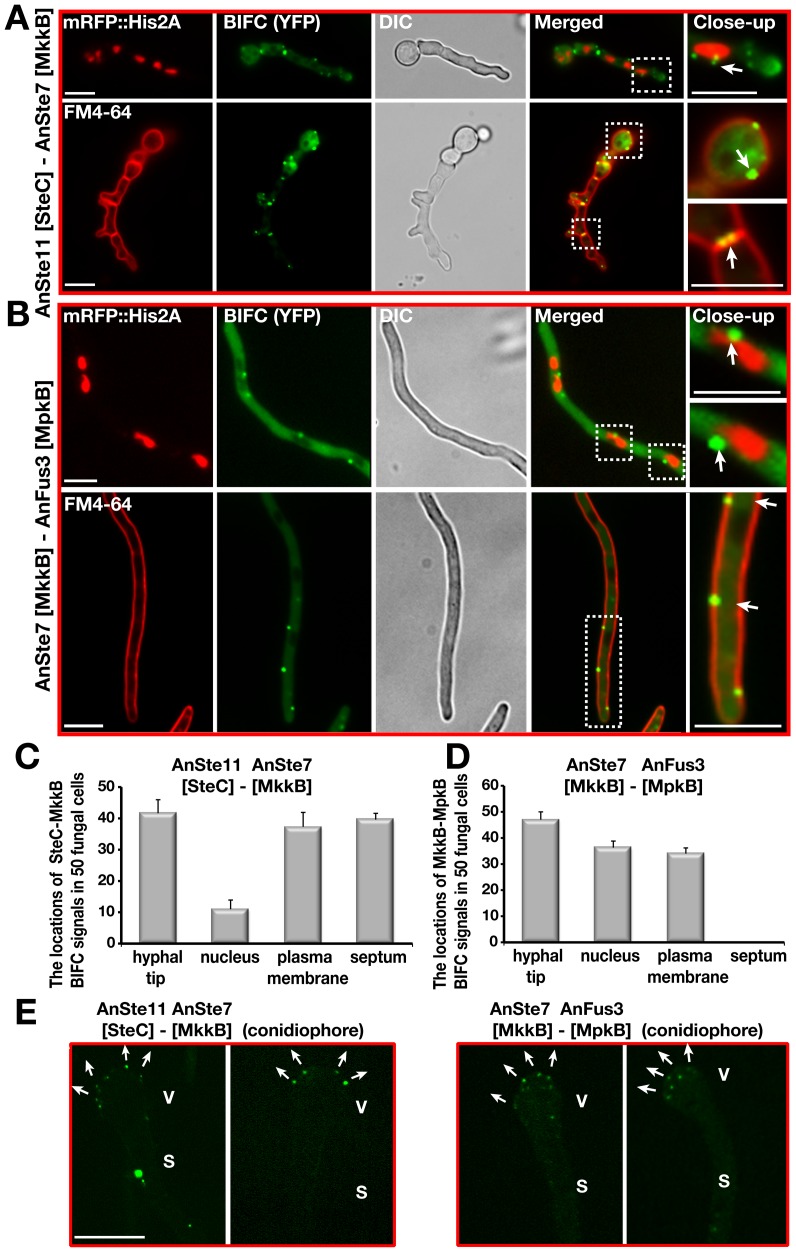
Confirmation of the subcellular interactions of the kinase complexes AnSte11-Ste7 and AnSte7-Fus3 by BIFC system. (A) Interaction of N-EYFP::AnSte11 [SteC] and C-EYFP::AnSte7 [MkkB] proteins in the hyphal cells. AnSte11-Ste7 kinase complexes are located at the plasma membrane, septal connections, the hyphal tip and partially nuclear envelope. The upper panel shows the localization of the YFP signal in comparison to nuclear mRFP::Histone2A fluorescence. Lower panel displays the YFP signal emitting cells stained with membrane dye FM4-64. (B) Physical interaction of N-EYFP::AnSte7 [MkkB] with C-EYFP::AnFus3 [MpkB] proteins in the fungal cells. (C) Quantification of the subcellular locations of the AnSte11-Ste7 complexes that are often present at the hyphal tip, plasma membrane, septum and nuclear envelope. N:50 fungal cells were counted in triplicate. Standard deviations are presented as vertical bars. (D) Subcellular locations of the AnSte7-Fus3 interactions. AnSte7-Fus3 complexes hardly localize to the septum and are found more on the nuclear envelope. (E) Assembly of the AnSte11-AnSte7 and AnSte7-AnFus3 complexes on the surface of vesicles of asexual conidiophores. Arrows indicate the growth directions of the metulae initials on the vesicles. V; vesicle, S; stalk. Size of the scale bars is 10 µm.

Bimolecular fluorescence complementation (BiFC) [38], [39] was applied to examine whether there are direct transient *in vivo* interactions between AnSte7 and Fus3, which could not be found by TAP purification (Figure 7B). Similar to the yeast localization of the Ste5-Ste11-Ste7-Fus3 MAPK module at the membrane, AnSte11-Ste7 and AnSte7-Fus3 interacted at the plasma membrane and also at the hyphal tip (Figure 7). There was an additional strong interaction of AnSte11-Ste7 at septa which border cellular segments as well as at septa of spore forming cells and spores (Figure 7C–7E). Quantification of the fluorescence intensity from the bright enhanced yellow fluorescent protein (EYFP) spots of AnSte11-Ste7, Ste7-Fus3 pairs revealed that they emit upto 10 fold more yellow fluorescence than the single EYFP molecules (Figure S6), suggesting that the kinase pairs form multimeric complexes.

**Figure 8:**
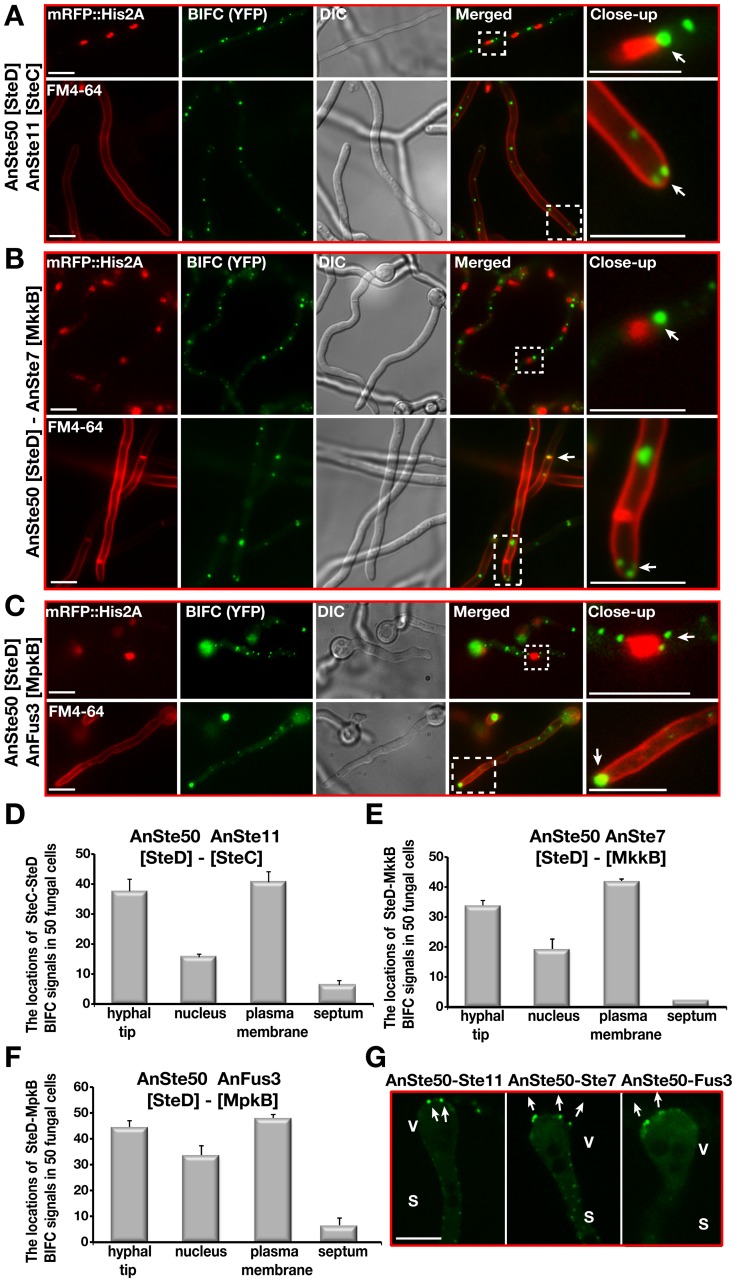
Subcellular locations of the AnSte50-Ste11, AnSte50-Ste7, and AnSte50-Fus3 complexes *in vivo*. (A) Interactions of N-EYFP::AnSte11 [SteC] and C-EYFP::AnSte50 [SteD] proteins in the fungal cells. Upper panel shows the nuclear mRFP signal and lower panel FM4-64 in comparison to the split YFP signal. (B) Interactions of AnSte50 [SteD] protein with AnSte7 [MkkB]. (C) Interactions of AnSte50 protein with AnFus3 [MpkB]. (D–F) Measurement of the subcellular locations of the AnSte50-Ste11, -Ste7, and Fus3 complexes that are frequently found in the nuclear envelope, plasma membrane, at the hypal tip and less often in the septal locations. Quantification was performed and analyzed as described in Figure 7. (G) Detection of the AnSte50-Ste11, AnSte50-Ste7, and AnSte50-Fus3 complexes on the vesicle (V) of the asexual conidiophore structures. S; stalk. Scale bars represent 10 µm.

Consistently to the yeast situation, the transcription factor AnSte12 as well as fungus specific factors VeA and LaeA specifically interacted with the MAPK Fus3 in the nucleus (Figure 1C, 1D). AnSte50 also interacted with the kinases at the plasma membrane and hyphal tip (Figure 8). Only AnSte11-Ste7 strongly interacted at the septa but there was hardly any interaction between AnSte7-Fus3 or between the AnSte50 and any of the kinases at the septa (Figure 7C and 7D, Figure 8D–8F).

### The entire MAPK module components migrate to the nuclear envelope to deliver AnFus3 into the nucleus

**Figure 9:**
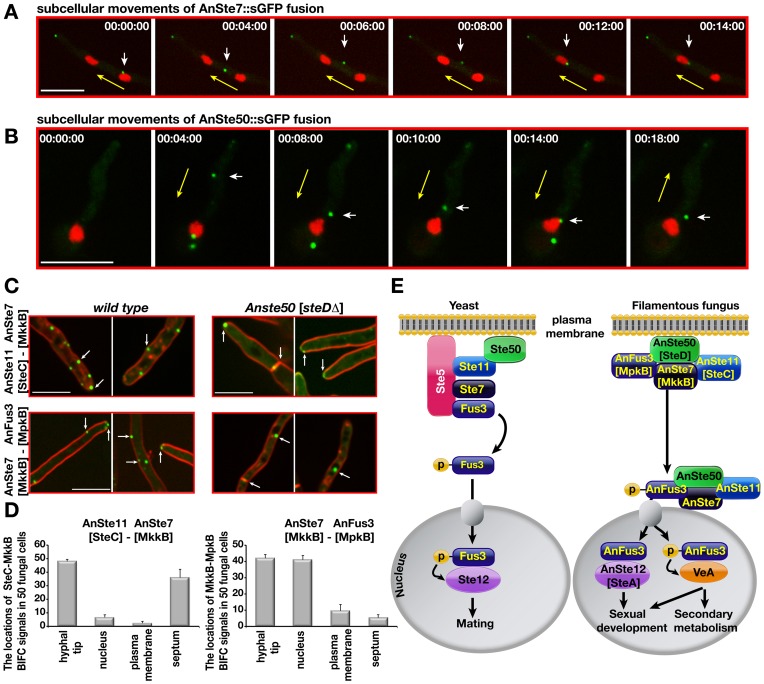
Cellular movements of AnSte7 and AnSte50::sGFP fusions and interactions of the AnSte11-Ste7, AnSte7-Fus3 complexes in the *steDΔ* mutant lacking AnSte50. (A) Cellular movements of the AnSte7::sGFP fusion. Single focal plane pictures of a time-lapse study for the AnSte7 fusion (in minutes) (Video S1). The MAPKK protein (white arrow) leaves the first nucleus; shortly after touching the membrane it reaches the second nucleus (visualized by RFP). The yellow arrow indicates movement direction. (B) AnSte50::sGFP cellular movement in both directions (Video S2). AnSte50 (white arrow) moves within the filament back to the nucleus (yellow arrow), and bounces back in the opposite direction after touching the nucleus. (C) Interactions of AnSte11-Ste7 complex occur at the hyphal tip, plasma membrane and the septa in wild type (see arrows). Membrane localization of AnSte11-Ste7 kinases drastically decreases in *Anste50* [*steD*Δ] strain. Hyphal tip and septa localizations are unaffected. AnSte7-Fus3 interact at the hyphal tip, membrane and partially nuclear envelope. (D) Quantification of the locations of the AnSte11-Ste7 and AnSte7-Fus3 complexes in *steD*Δ strain. N:50 fungal cells were counted in triplicates. Standard deviations were given as vertical lines. Scale bar, 10 µm. (E) A comparative depiction of the MAPK modules and their action in the single-cell yeast and filamentous fungus. In the yeast system, (MAP3K)Ste11-(MAP2K)Ste7-(MAPK)Fus3 kinase complex assembles on the scaffold protein Ste5 which tethers the complex close to the plasma membrane. Ste50 is additionally required for membrane recruitment of the Ste11. Activation of Fus3 by Ste7 phosphorylation results in entry of the active Fus3 into the nucleus where it phosphorylates Ste12 transcription factor for mating responses. In the filamentous fungus, AnSte50 is partly responsible for the membrane attachment of the AnSte11-Ste7-Fus3 complex, which migrates to the nuclear envelope presumably to keep the AnFus3 active. Finally, AnFus3 (MpkB) is released into the nucleus where it interacts with SteA (Ste12) for hyphal fusions and sexual development. It also phosphorylates the velvet A protein, which in turn leads to activation of secondary metabolism with development.

Yeast Ste7-Ste5-Fus3 migrates to tips of mating projections in pheromone treated cells. Only Fus3 travels to the nucleus upon activation by Ste7 [13]. *A. nidulans* is a homothallic fungus, which does not require a mating partner. Time lapse images revealed that MAPK module components AnSte7 and Ste50::sGFP can move within the fungal cell along the membrane. During the cellular movements, these molecules shortly touched the membrane then hit the nucleus. Sometimes, fusion protein moved back after contacting the nucleus in the opposite direction. (Figure 9A and 9B, Videos S1 and S2).

The dynamics of the protein interactions of the BiFC expressing strains were further analysed by time lapse movies (Videos S3, S4, S5, S6). The AnSte7-Fus3 pair moved together along the plasma membrane (Video S3, Figure S7A) towards the first nucleus, then they advanced to the next nucleus while some other spots did not move distinctly. Likewise, AnSte50-Fus3 complexes left one nuclear envelope, touched the membrane and moved to the next nucleus (Video S4, Figure S7B). Similar movements were also observed for other complexes of the MAPK module (not shown). AnSte11-Ste7 can dissociate from the plasma membrane, cross the cytoplasm and reach the nuclear envelope (Videos S5 and S6, Figure S7C, S7D).

The major difference to the yeast situation is that the MAPK module of *A. nidulans* travels from the outer border of the fungal cell through the cytoplasm to the nuclear envelope. The AnSte7-Fus3 pair as well as pairs of AnSte50 with all three kinases interacted at the nuclear envelope (Figure 7 and Figure 8). These data suggest significant differences in the molecular mechanism how a MAPK signal is transmitted in yeast in comparison to a filamentous fungus.

### AnSte50 is required for efficient membrane attachment of MAPK complexes

The interactions of the AnSte11-Ste7 and AnSte7-Fus3 complexes were examined in *steD*Δ strain to examine AnSte50 function for cellular location of the module. The interaction of the three kinases at the plasma membrane of wild type (Figure 7) was abolished for AnSte11-Ste7 and drastically reduced for AnSte7-Fus3 in the *steD* mutant (Figure 9C, 9D). Plasma membrane localizations of the AnSte7 and Fus3::sGFP fusions were also reduced in the *steD* mutant (not shown). Contrastingly, the localization of the entire module at the hyphal tip or for the partial module AnSte11-Ste7 at the septum seems to be mediated by a mechanism which is largely independent of AnSte50.

These data suggest that AnSte50 supports association of the *A. nidulans* MAPK module with the plasma membrane but it does not affect the hyphal tip and septum localizations.

## Discussion

We describe here the *A. nidulans* Fus3 MAPK module which is involved in sexual development and the control of secondary metabolism and releases AnFus3 into the nucleus. Our data suggest a provocative additional hypothesis: AnFus3 is able to travel along the membrane and to cross the cytoplasm to the nuclear envelope in complexes with AnSte7 MAP2K, AnSte11 MAP3K and the adaptor protein AnSte50. In the nucleus AnFus3 interacts with transcription factor AnSte12 for sexual development. The additional interaction of AnFus3 with VeA or yet unidentifed targets may promote VeA-VelB formation which is required for coordinated development and secondary metabolism (Figure 9E). The *A. nidulans* Fus3 MAP kinase module is preferentially assembled at distinct intracellular locations, such as the hyphal tip, the septa, the plasma and nuclear membranes. Membrane localisation of the module is presumably relevant to perceive external signals as in yeast. Sexual development is defective when membrane localization of the module is impaired as in strains without intact AnSte50. Tip localisation could be important for hyphal fusions and cell-cell contacts. MAPKK AnSte7 and MAP3K AnSte11 but not other components interact at septa suggesting additional phosphorylation functions at septa independent of AnFus3. Corresponding mutants displayed strong deformations in the septa between developing asexual spores and spore forming cells but did not show any abnormal septation pattern in vegetative hyphae (not shown). This suggests a possible additional link between kinases of the module and regulators of asexual development.

Intracellular distances in a filamentous fungus are significantly larger than in yeast. Several steps can be distinguished for signal transduction from surface to nucleus of *A. nidulans*. (i) From hyphal tip to plasma membrane: AnSte50 is primarily required for efficiently anchoring the MAPK module to membranes, but not to hyphal tips. AnSte50 might also contribute like in yeast to Ste11 MAP3K activation. The essential function of AnSte50 for signal transduction is supported by the defect of sexual development and lack of AnFus3 phosphorylation in a *steD* mutant. The AnSte50 independent localization at the hyphal tip suggests an additional yet unknown anchoring function for the AnFus3 module at the hyphal tip. The anchoring mechanism could include small membrane bound vesicles at the *Spitzenkörper* which could explain some of our localization results (Figure 6B, Figure 7, Figure 8).

The lack of AnSte11 did not cause any changes in the subcellular localization of AnSte7, indicating that AnSte11 is not required for proper AnSte7 localization. The lack of AnSte50 had a drastic effect on the localization of MAPK module complexes. AnSte50 interacts with all components of the MAPK module and might provide a binding platform for the other MAPK components which even works when AnSte11 is absent (Figure 8).

(ii) In yeast Fus3 dissociates from the Ste5 tethered pheromone pathway module and enters into the nucleus [13]. Transport of the AnFus3 in the AnSte50-Ste11-Ste7 complex (or subcomplexes) to the nuclear envelope as additional signal transmission step in *A. nidulans* might secure that AnFus3 can be kept active over larger distances until it finally reaches the nucleus. It will be interesting to analyse phosphorylation states of kinases at different cellular locations during signal transduction.

(iii) Import of AnFus3 from nuclear envelope into nucleus: AnFus3 presumably dissociates from the kinase module at the nuclear envelope in a mechanism wihich is unknown. After entry into the nucleus, AnFus3 interacts with AnSte12, and presumably phosphorylates it. AnFus3 phosphorylates the velvet protein VeA, which efficiently associates with VelB and LaeA. It is yet unclear whether there are additional AnFus3 targets which support VelB-VeA complex formation. VelB-VeA then contributes with AnSte12 to sexual fruiting body development and the trimeric VelB-VeA-LaeA concomitantly promotes expression of distinct genes for secondary metabolites (Figure 9E). These include the production of the mycotoxin sterigmatocystin or antitumor agent terrequinone but not the antibiotic penicillin synthesis.

The MAPK module of *A. nidulans* is presumably involved in integrating multiple signals and enabling an adequate cellular response. Oxylipins represent currently the only known pheromones of Aspergilli but the receptors are unknown [40]. In yeast nitrogen starvation induces the same kinase module as pheromones, and part of the components are also involved in response to osmotic stress. It is likely that the AnSte50-Ste11-Ste7-Fus3 and the septal AnSte11-Ste7 modules have additional targets other than AnSte12 and VeA, which remain to be identified.

An interesting open question is whether other organisms also transport their Fus3 MAPK counterpart together with the entire module from surface to nuclear envelope. This results in questions about transport control points and module attachment sites on the nuclear envelope where future work in *A. nidulans* could deepen insights into the molecular mechanism of information transfer through the cell.

## Materials and Methods

### Strains, media, transformation, and cultivation of the microorganisms

Fungal strains created and used in the course of this study are given in Table S7. *Aspergillus nidulans* strains; FGSCA4 (*veA*+), TNO2A3 (*veA1*) [41], AGB152 (*veA*+) [42], AGB154 (*veA*+), AGB506 (*veA*+) [31], AGB551 (*veA*+), AGB552 (*veA*+) served as wild type transformation hosts for the knock-out, epitope tagging, BIFC, and overexpression experiments. Further details for the strains transformed with various plasmids are given in Table S8. Culturing fungal strains were described in detail elsewhere [43]. DH5α and MACH-1 (Invitrogen) *Escherichia coli* strains were applied for recombinant plasmid DNA. *Aspergillus* and *E. coli* strains were cultured as described previously [30]. Fungal and bacterial transformations were carried out as given in detail [30].

### Manipulation of nucleic acids

Circular and linear DNA molecules were created based on the standard recombinant DNA technology protocols in detail [30]. Plasmids and oligonucleotides applied and constructed in this study are given in Table S8 and Table S9. During polymerase chain reaction (PCR) different kind of DNA polymerase combinations including *Pfu* (MBI Fermentas), *Phusion* (Finnzymes), *Platinum-Taq* (Invitrogen) and *Taq* (Fermentas) were used. Linear and circular DNA constructs were created as given below.

### Creation of *Anste7* [*mkkB*] deletion cassette and complementation plasmid

For construction of *Anste7* [*mkkB*] deletion fragment, 1.1 kb 5 UTR and 0.6 kb 3 UTR flanking regions of AN3422 locus were amplified with 3422-A/C and 3422-D/F, respectively. These two fragments were fused with *ptrA* marker (amplified from pPTRII) by fusion PCR (3422-B/E), creating 3.6 kb deletion fragment which was transformed into TNO2A3 (*veA1*), AGB154 (*veA*+), creating AGB586, AGB587 strains, respectively. Complementation plasmid pME3854 was constructed by amplifying 4.2 kb genomic *Anste7* [*mkkB*] locus (Comp-A/B) and subsequent cloning into *Stu*I site of pAN8-1 [44] plasmid carrying the phleomycin resistance marker. Deletion and complementation events were verified by the Southern hybridization (Figure S8A–S8B).

### Overexpression of *Anste7* [*mkkB*]

Primers OZG302/303 amplified the 1.6 kb cDNA of *Anste7* [*mkkB*] from sexual cDNA library [45]. T4 Polynucleotide kinase (PNK) treated phosporylated amplicons were inserted into *Pme*I site of pME3160 [30] under nitrogen source inducible *niiA* promoter leading to pME3855 that was transformed into AGB152, which resulted in AGB662.

### Generation of endogenous *Anste7* [*mkkB*]*::gfp* and *ctap* gene replacement modules

For the purpose of substitution of the orinigal *Anste7* [*mkkB*] locus by *mkkB::gfp* and *ctap*, *mkkB* promoter including *mkkB* ORF (2.85 kb) and terminator regions (0.6 kb) were PCR-amplified from genomic DNA (3422-A/OZG380 for *gfp*, 3422-A/OZG382 for *ctap*, and OZG314/3422-F). Finally, the fragments 3422-A/OZG380 and OZG314/3422-F were fused to *sgfp::natR* module (with oligos 3422-B/3422E) creating 5.4 kb *mkkB::sgfp::natR* fusion construct. Likewise, 3422-A/OZG382 and OZG314/3422-F, and *ctap::natR* were joined by fusion PCR (3422-B/3422E) resulting in *mkkB::ctap::natR* cassette for gene replacement. *mkkB::sgfp::natR* construct (5.2 kb) was transformed into TNO2A3 and SWH51 [24], which yielded AGB590 and 592, respectively (Figure S8D–S8E). Similarly, *mkkB::ctap::natR* was introduced into AGB551 and SWH51 resulting in AGB597 and 598.

### Construction of Bimolecular Fluorescence Complementation (BIFC) plasmids

*Anste11* [*steC*] ORF was amplified from gDNA (OZG389/OZG392) and fused to *nyfp* (OZG73/387) leading to *nyfp:*:*Anste11* [*steC*] fusion fragment which was cloned in *Pme*I site of pME3160 plasmid yielding pME3859 (*nyfp::steC*). *Anste7* [*mkkB*] ORF was PCR-amplified (for *nyfp*, OZG389/303, for *cyfp* OZG390/303) from genomic DNA followed by fusion to *nyfp* and *cyfp*, which produced *nyfp::Anste7* [*mkkB*] and *cyfp::Anste7* [*mkkB*] fusions. Similar to *Anste11* [*steC*] cloning, *nyfp::mkkB* fragment was inserted in *Pme*I site of pME3160 generating pME3861 plasmid.

To test AnSte11/AnSte7 interaction, *cyfp::mkkB* was cloned in *Swa*I site of pME3859 leading to pME3860 that was brought in AGB506, which generated AGB599. For AnSte7/AnFus3 interactions, *Anfus3* [*mpkB*] cDNA was amplified from cDNA library (OZG404/403 for *nyfp*, OZG405/403 for *cyfp*). OZG404/403 and OZG405/403 were fused to *nyfp* (OZG73/387) and *cyfp* (OZG75/388) fragments yielding *nyfp::mpkB* and *cyfp::mpkB*. *nyfp::mpkB* fragment was inserted in *Pme*I site of pME3160, which led to pME3864 and *cyfp::mpkB* was cloned in *Swa*I site of pME3861 generating pME3862 (*nyfp::Anste7* [*mkkB*]/*cyfp::Anfus3* [*mpkB*]). The BIFC plasmid pME3862 was introduced in AGB506 in order to generate AGB600. *Anste12* [*steA*] gDNA was amplified with oligos OZG400/401 and fused to *cyfp* by fusion PCR (OZG75/400).

*cyfp::veA*, *cyfp::velB*, *cyfp::vosA* and *cyfp::laeA* were produced as described in detail [31]. Insertion of *cyfp::steA*, *cyfp::veA*, *cyfp::velB*, *cyfp::vosA* and *cyfp::laeA* in *Swa*I site of pME3864 yielded following plasmids pME3865 (*mpkB*/*steA*, AGB601), pME3866 (*mpkB/veA*, AGB623), pME3867 (*mpkB*/*velB*, AGB625), pME3868 (*mpkB*/*vosA*, AGB624), pME3869 (*mpkB*/*laeA*, AGB622).

*Anste50* [*steD*] cDNA was amplified from cDNA library (OZG500/501) and joined to *cyfp* fragment with oligos OZG75/OZG500. Finally, this fragment was cloned in *Swa*I sites of pME3859, 3861, 3864, leading to plasmids pME3870, 3871 and 3927, respectively.

### Generation of *Anste50* [*steD*] deletion, endogenous *steD::sgfp*, and *steD::ctap* replacement fragments

*steD* deletion, *steD::sgfp* and *steD::ctap* linear fragments were created in an identical manner to *mpkB* constructs. *steD* deletion; OZG470/472, *ptrA*, OZG473/475 were fused by using oligos OZG471/474 in a fusion PCR. *steD::sgfp::natR* (5.1 kb); OZG470/564, *sgfp::natR*, OZG566/475 were joined by oligos OZG471/474. *steD::ctap::natR* (4.9 kb); OZG470/565, *ctap::natR*, OZG566/475 were joined by oligos OZG471/474. *steD* deletion cassette was brought into AGB552 and 551 generating, AGB576 and 650, respectively. *steD::sgfp* and *steD::ctap* were used for gene replacement in AGB551, giving rise to AGB657 and 659, respectively (Figure S9A–S9C).

### Construction of *Anfus3* [*mpkB*] deletion, *sgfp, ctap*, and *mrfp* fusions

*mpkBΔ::ptrA* deletion fragment was constructed by amplification of 1.2 kb 5 and 3 flanking regions of *mpkB* with primers (for 5 UTR, OZG443/445, for 3 UTR OZG446/448). OZG443/445, *ptrA* marker, and OZG446/448 were fused by oligos OZG444/447 creating 3.9 kb *mpkBΔ::ptrA* construct. Consequently, *mpkBΔ::ptrA* fragment was transformed into AGB552, which generated AGB611. To create *Anfus3* [*mpkB*]*::sgfp* and *ctap* linear fragments, *mpkB* promoter as well as ORF was amplified by (OZG443/560 for *gfp* fusion and OZG443/561 for *ctap* fusion). OZG443/560, *gfp::natR*, and OZG562/448 were co-fused by oligos OZG444/447 creating 5.3 kb *mpkB::sgfp::natR* fragment. OZ443/561, *ctap::natR*, and OZG562/448 linear DNAs were fused to make *mpkB::ctap::natR* gene replacement fragment (5.1 kb). *mpkB::sgfp* and *mpkB::ctap* were transformed into the AGB551 strain, which yielded AGB654 and 659, respectively (Figure S9D–S9G). For creation of constitutively expressed *mpkB::mrfp* fusion, *gpdA* promoter (OZG735/736), *mpkB* cDNA (OZG737/738), and mRFP::H2A terminator (OZG739/740) were amplified and fused together (OZG735/740). The fusion was cloned in the *Swa*I site of a *pyrG* marker bearing plasmid. The final plasmid pME3966 was introduced into AGB590 and AGB657 for co-localization studies.

### Construction of the yeast complementation plasmids

Promoter and terminator regions of *STE7* (promoter OZG679/680, terminator OZG683/684) and *FUS3* (promoter OZG685/686, terminator OZG689/690) were amplified from the wild type yeast genomic DNA. These regions were either fused to the gDNA or cDNAs of *Anste7* [*mkkB*] (681/682) and *Anfus3* [*mpkB*] (687/688) genes. Resulting fusions *^pro^STE7::mkkB* gDNA*::STE7^ter^*, *^pro^STE7::mkkB* cDNA*::STE7^ter^*, *^pro^FUS3::mpkB* gDNA*::FUS3^ter^* and *^pro^FUS3:: mpkB* cDNA*::FUS3^ter^* were cloned in *Sma*I site of yeast centromeric plasmid pRS316 (*URA3*) [46] yielding pME3958, 3959, 3960 and 3961, respectively. *STE7* (OZG679/684), *FUS3* (OZG685/690), and *KSS1* (OZG691/692) genomic loci were amplified and similarly cloned into *Sma*I site of pRS316 resulting in pME3962, 3963, and 3964 plasmids, respectively. These control and chimeric constructs were transformed into the appropriate *ste7*, *fus3*, and *fus3*/*kss1* [47] deletion strains.

### Hybridization techniques and analysis of nucleic acids

Southern and Northern hybridizations were carried out as explained in detail [30], [43] according to protocols [48], [49].

### Immunoblotting

Immunoblotting experiments for recognition of GFP, TAP fusion, VeA, and actin in protein extracts was performed according to described protocols [31]. α-phospho 44/42 (4377, Cell Signaling Technology Inc) was used for detection of the phosphorylated AnFus3 [MpkB]. For the detection of the phosphorylated proteins, α-phosphoserine/threonine (ab17464, Abcam) was employed. Manufacturers protocols were followed for incubation times and buffer applications of phosphospecific antibodies.

### Expression of recombinant proteins

Proteins were expressed in Rosetta 2 (DE3) using ZYM5052 [50] media supplemented with 30 µg/ml Chloramphenicol and 100 µg/ml Ampicillin (GST-LaeA91) or 30 µg/ml Kanamycin (Velvet proteins) at 16°C. Cells were harvested by centrifugation, resuspended in lysis buffer (30 mM HEPES pH 7.4, 400 mM NaCl, 30 mM Imidazol) and lysed by passing through a Microfluidics Fluidizer at 0.55 MPa. The lysate was cleared by centrifugation at 30000×g for 30 minutes. His-tagged proteins were purified with a 5 ml NiNTA-Sepharose (GE Healthcare) and GST-tagged LaeA91 with a 5 ml GSH-Sepharose (GE Healthcare) column connected to an ÄKTA Prime chromatography system. After washing with 10 column volumes with lysis buffer, proteins were eluted with elution buffer plus 400 mM Imidazol or 30 mM reduced Glutathione. Velvet proteins were desalted with a HiPrep Desalting 26/10 column (GE Healthcare) into storage buffer (10 mM HEPES pH 7.4, 400 mM NaCl). GST-LaeA91 was cleaved with PreScission Protease at 4°C for 16 h and further purified by gel-filtration using a Superdex 200 26/60 and a final 5 ml GSH-Sepharose column both equilibrated in gelfiltration buffer (10 mM HEPES pH 7.4, 150 mM NaCl). All proteins were shock-frozen in liquid nitrogen and stored at −80°C until further use.

### Protein immunoprecipitation

In order to immunoprecipitate GFP fusion proteins, protein crude extracts were prepared from vegetatively grown cultures. 100 µl GFP-Trap sepharose (Chromotek) was washed twice with 1 ml protein extraction buffer (50 mM Tris pH 7.5, 100 mM KCl, 10 mM MgCl_2_, 0.1% NP40, 10% Glycerol, 20 mM β-glycerophosphate, 2 mM Na_3_VO_4_, 5 mM NaF, 0.5 mM PMSF, 1 mM benzamidine, 1 mM EGTA, 1 mM DTT). 20 ml (150 mg total) protein crude extract was incubated with 100 µl GFP-Trap sepharose (Chromotek) at 4°C for 2 hours on a rotating platform. Afterwards, sepharose-extract mixture was centrifuged at 4000 rpm at 4°C for 1 min. Crude extract was removed with a 5 ml pipette. The sepharose was washed twice with 20 ml of protein buffer and centrifuged at 4000 rpm at 4°C for 1 min. This step was repeated one more time. Finally, 1 ml of protein buffer was added and the sepharose was resuspended. Each of the 200 µl sepharose buffer mixture was transferred into 1.5 ml eppendorf cups and centrifuged at 4000 rpm at 4°C for 1 min and supernatant was removed. Immunoprecipitated proteins were washed three times with 1 ml kinase reaction buffer (KRB; 20 mM Tris pH 7.5, 10 mM MgCl_2_, 1 mM DTT, 1 mM benzamidine, 1 mM Na_3_VO_4_, 5 mM NaF, 0.1 µCi [^32^P]-ATP).

### *In vitro* phosphorylation and dephosphorylation assay

*In vitro* phosporylation assay was performed with modifications according to protocol given in [51]. For *in vitro* phosphorylation experiment, 30 µl KRB, containing 0.1 µCi [^32^P]-ATP and 10 µg recombinant protein were added to the sepharose beads and incubated at 30°C for 35 minutes with the periodic resuspensions in every five minutes. Afterwards, reaction tubes were centrifuged at 4000 rpm at R/T for 1 min and supernatants containing phosphorylated proteins were transferred into new eppendorf cups. Supernatans and sepharose containing immunoprecipitated proteins were mixed with 3× protein loading dye (30 µl supernatant and 15 µl loading dye) and incubated at 95°C for 10 min. 30 µl of the supernatant fraction was run on 4–15% gradient SDS gel that was dried for 2 h and exposed to Kodak X-omat film for 5 hours. 10 µl of the reaction was used for visualization of the proteins with coomassie staining. 2 µl of sepharose was used for immunoblotting and ponceau staing for validation of equal immunoprecipitated target protein (MpkB or GFP). For non-radioactive kinase experiments, same KRB buffer containing 5 µM ATP was used. Supernatants were treated with 1000 units lambda protein phosphatase (New England Biolabs) in the presence of 1 mM MnCl_2_ at 30°C for 1 hour. Samples were added with 3× loading dye and boiled at 95°C for 10 min. 3 µl of the samples were used for immunoblotting.

### Tandem Affinity Purification (TAP) protocol and LC-MS/MS protein identification

For the TAP purification of the MkkB, MpkB, SteD, and VeA interacting proteins and further LC-MS/MS identification previously published protocols were applied [30].

### Confocal spinning disc and fluorescence microscopy

*A. nidulans* strains expressing various fluorescence proteins (EYFP/sGFP/mRFP) were inoculated in the 8-well borosilicate coverglass system (Nunc) containing the liquid minimal medium. Widefield fluorescence photographs were taken with an Axiovert Observer. Z1 (Zeiss) microscope equipped with a CoolSNAP ES2 (Photometrics) digital camera. CSU-X1 A1 confocal scanner unit (Yokogawa) connected with QuantEM:512SC (Photometrics) digital camera was used for laser microscopy. The SlideBook 5.0 software package (Intelligent Imaging Innovations) was used for fluorescence and laser confocal image and movie recording as well as productions. We defined signals as plasma-membrane localized if we found the signals that are at the border of the silhouette of the fungal cell or even surmount the fungal cell; similarly, we defined signals as nucleus-associated when we found multiple signals at the border of the nuclear silhouette.

### Quantification of the YFP fluorescence

The EYFP protein was purified by using GFP-Trap as described for GFP protein. EYFP molecules were allowed to attach to poly-L-lysine coated coverslips for 10 minutes, in PBS buffer. Fungal cultures were grown as described above. The preparations were imaged using a SP5 TCS STED microscope (Leica Microsystems), under 514 nm excitation (provided by an Argon laser), using a 100× oil-immersion objective (1.4 NA, Leica). The images were processed by a custom-written routine in Matlab (The Mathworks Inc.). Briefly, the spots were identified by the application of an automatic threshold based on the intensity of the background. We then used Gaussian fits to the spots to determine their intensity, and to correct for the background intensity, which provided the baseline of the fits.

### Analysis of secondary metabolites

Extraction of sterigmatocystin (ST) and thin layer chromatography (TLC) was carried out as given in detail [43]. Penicillin levels were determined as published previously [52].

## Supporting Information

Figure S1 Functionality of AnFus3 [MpkB] and AnSte50 [SteD]::sGFP and TAP fusions for fungal growth, sexual development. (A) Phenotypes of the wild type, *Anfus3* [*mpkB*Δ], *Anste50* [*steD*Δ], *Anfus3* [*mpkB*]*::sgfp*, *Anfus3* [*mpkB*]*::ctap*, *Anste50* [*steD*]*::sgfp*, *Anste50* [*steD*]*::ctap* strains incubated under dark and light conditions for 5 days at 37°C. Black frames are the stereomicroscopic images of the colonies on the plates. Mature fruiting bodies are indicated by yellow arrows. *Anfus3* [*mpkB*Δ] and *Anste50* [*steD*Δ] strains cannot produce mature cleistotheica (indicated by red arrows) instead form nest-like structures. (B) Quantification of the asexual conidiations of the strains from (A). Reduced asexual sporulation seen in *Anfus3* [*mpkB*] and *Anste50* [*steD*] mutants. Replacement strains sporulate more efficient than the deletion strains. (C) Expression of AnSte50 [SteD] and AnFus3 [MpkB]::sGFP proteins during different developmental stages (vegetative, asexual and sexual, respectively). 68 kDA AnFus3 [MpkB] and 81 kDA AnSte50 [SteD]::sGFP fusion proteins were detected by α-gfp. Constitutive transcript levels of the *mpkB* and *steD* genes from the same experiments. Actin levels served as loading control for immunoblotting (80 µg in each lane), and internal *gpdA* expression was used as control for Northern hybridizations. 20 µg RNA was loadeded in each sample.
(TIF)

Figure S2 Transcript levels of *velB* and *laeA* and cellular localizations of the velvet complex components in the wild type and *Anste7* [*mkkB*Δ] strain. (A) Transcript levels of *velB* and *laeA* in the wild type and *mpkB* mutant background. RNA levels of *velB* and *laeA* from two different time points (24 and 72 hours) were quantified and normalized to the internal control gene expression *gpdA*. *velB* levels do not change significantly, but *laeA* transcript is drastically downregulated. Black bars represent standard deviations. (B) Localization patterns of VeA, VelB and LaeA::sGFP fusions in the wild type and *mpkB* mutant background. Fungal strains were grown in the darkness for 24 hours at 30°C and pictures were taken in a fluorescence microscope. Scale bars represent 10 µm.
(TIF)

Figure S3 Global alignment of the AnSte7 [MkkB] of *Aspergillus nidulans* with other eukaryotic MAPK Kinase homologs. *A. nidulans* AnSte7 [MkkB] (ANID_03422) was aligned with the amino acid sequences from *Aspergillus fumigatus* Afu3g05900, *Neurospora crassa* MAPK Kinase (NCU04612), *Magnaporthe oryzae* (EHA48601.1), *Podospora anserina* (XP_001910826.1), *Trichoderma atroviride* (EHK42325.1), *Coccidioides immitis* (XP_001246770.1), *Saccharomyces cerevisiae* Ste7p, and *Homo sapiens* MAPK2K1. Conserved protein kinase domains and the central part showing higher similarity are indicated with red rectangle. N- and C-terminal sequences of the kinase proteins show less similarity. *S. cerevisiae* and *H. sapiens* proteins often break the alignment. Filamentous fungus kinases show higher similarity to the AnSte7 protein.
(TIF)

Figure S4 Increased sexual development caused by overexpression of *Anste7* [*mkkB*] gene and functional complementation assays in yeast. (A) Growth of the control (empty plasmid carrying strain) and *Anste7* [*mkkB*] OE (under *niiA* promoter) strains under white light (90 µWm^2^) and dark conditions on repressing (NH_4_^+^) and inducing (NO_3_^−^) media. Lower panel shows the plate pictures, upper squares are the stereomicroscopic images taken from the plates. 1×10^4^ spores were point-inoculated and grown at 37°C for 5 days. (B) Validation of *Anste7* [*mkkB*] overexpression by Northern blot. *gpdA* transcript levels and rRNA were used as equal loading controls. Total 20 µg RNA was applied in each lane. (C) Quantification of cleistothecia production from (A). Increased cleistotheica production in *Anste7* [*mkkB*] OE strain in the dark on nitrate containing inducing medium. Vertical lines are the standard errors originating from different counts. L; light, D; dark. Rep; repressed, Ind; induced. (D) Either cDNA or ORF of *Anste7* [*mkkB*] and *Anfus3* [*mpkB*] expressed under yeast *STE7* or *FUS3* promoters in a centromeric self-replicating plasmid. These constructs were expressed in the respective *fus3*, *ste7* and *fus3*/*kss1* double mutants. Strains were grown in the presence of 15 µg alpha factor given on the paper discs at 30°C for 3 days. Alpha factor in wild type (empty plasmid) and complementation strains (*STE7* in *ste7* mutant, *FUS3* in *fus3* mutant, *FUS3* in *fus3*/*kss1* mutant) results in a strong growth inhibition (halo). *ste7* and *fus3*/*kss1* mutants do not show any response to the pheromone treatment. *fus3* mutant exhibits a reduced response (cloudy halo). AnSte7 and Fus3 do not remediate the halo phenotype of the *ste7* and *fus3* mutants. *mpkB* cDNA partially restores the pheromone response of the *fus3*/*kss1* double mutant.
(TIF)

Figure S5 Functionality of the AnSte7 [MkkB]::sGFP and cTAP tag fusions for fungal growth and sexual development. (A) Development of the wild type, *Anste7* [*mkkB*]*::sgfp*, *Anste7* [*mkkB*]*::ctap*, *Anste7* [*mkkB*Δ] strains on plates incubated under white light (90 µWm^2^). *Anste7* [*mkkB*]*::sgfp* and *ctap* look like the wild type strain. (B) Conidia production capacities of the strains from (A). Strains carrying *Anste7* [*mkkB*]*::sgfp* and *ctap* constructs produce similar levels of conidia of the wild type levels. Vertical bars are the standard deviations of quantifications. Same spore number was used for inoculation as in Figure S4. (C) AnSte7 [MkkB] protein levels under native promoter during different developmental stages of *A. nidulans*. Strains were grown vegetatively 20 h, and transferred on plates and incubated under light conditions (2, 6, 12, 24 hours) for asexual development and dark for sexual development. Protein undergoes degradation during 24 h asexual and sexual time points. *Anste7* [*mkkB*] transcripts are expressed constitutively during different stages. Actin levels and ethidium bromide stained ribosomal RNA served as loading controls.
(TIF)

Figure S6 Quantification of the EYFP fluorescence intensities from single EYFP and AnSte11-Ste7 and AnSte7-Fus3 BIFC complexes. (A) The intensity of the spots produced by the AnSte11-Ste7 and AnSte7-Ste3 complexes were measured (see Figure 7 for examples). The intensities are comparable, although the values tend to be higher for AnSte11-Ste7 (blue) than for AnSte7-Fus3 (red). To obtain an estimate of the number of molecules in the complexes, these values were compared to the intensity of single EYFP molecules attached to coverglasses (green). (B) The bar graphs indicate the average values for the intensities (obtained from the same datasets as in A). The intensity of the complexes is ∼9-fold (AnSte7-Fus3) or ∼10-fold (AnSte11-Ste7) higher than that of single EYFP molecules, suggesting the presence of 9 to 10 molecules in a complex. The bars show the mean and standard error; 50–300 spots were analyzed for each condition.
(TIF)

Figure S7 Spatial movements of the AnSte7-AnFus3, AnSte50-AnFus3, AnSte11-AnSte7 binary complexes within the fungal cells. (A) Movement of the AnSte7 [MkkB]-AnFus3 [MpkB] complex (white arrow) that touches the nucleus during intracellular translocations (17 min) (Video S3). Yellow arrows indicate the direction of the movements. (B) A movement of the AnSte50-AnFus3 complexes between two nuclei. Complex leaves the first nucleus, and slightly touches the membrane (a small deviation to upper side) reaches to the second nucleus (Video S4). (C) A horizontal backwards movement of the AnSte11 [SteC]-AnSte7 [MkkB] complexes from hyphal tip. Complexes touch the nucleus during bypass (Video S5). (D) A vertical movement of the AnSte11-AnSte7 complexes from membrane to the nuclear envelope (Video S6). White arrows indicate the YFP spots representing the binary complexes moving to the nucleus.
(TIF)

Figure S8 Southern hybridizations for the gene replacement experiments involving *Anste7* [*mkkB*] locus. (A) A comparative depiction of the genomic *Anste7* [*mkkB*] (AN3422) and the deletion of the locus by the selection marker pyrithiamine resistance gene, *ptrA*. Blue bar represents the Southern probe used in hybridizations. (B–C) Southern hybridization results of the *mkkB* deletion and complementation strains. Sizes of the restriction bands confirm the gene replacement and ectopic complementation of the knock-out strain by the complementation plasmid. Sizes of the restriction fragments are given in base pairs. (D) Schematic drawings of the *Anste7* [*mkkB*] locus gene replacements by *Anste7* [*mkkB*]*::sgfp::natR* and *Anste7* [*mkkB*]*::ctap::natR*. The cutting sites of the common restriction enzymes are indicated in the theoretical maps. (E–F) Southern results of the *Anste7* [*mkkB*]*::sgfp::natR* and *Anste7* [*mkkB*]*::ctap::natR* strains in comparison to the wild type locus. Bands released by restriction digests are in agreement with the theoretical maps of the replaced loci.
(TIF)

Figure S9 Verification of the gene replacements for *Anste50* [*steD*] and *Anfus3* [*mpkB*] loci. (A) Common restriction enzyme cutting maps of the wild type *Anste50* [*steD*] (AN7252) locus, *steD*Δ*::ptrA*, *Anste50* [*steD*]*::sgfp::natR*, and *Anste50* [*steD*]*::ctap::natR* gene replacements. Blue lines show the probe binding sites during Southern hybridizations. (B–C) Southern hybridizations of gene replacements in comparison to the wild type *Anste50* [*steD*] locus. Restriction enzymes used during Southern hybridizations are shown at the top of the blot. Lengths of the restriction fragments are given in base pairs. (D) Restriction map of the *Anfus3* [*mpkB*] (AN3719) locus and corresponding gene replacements for deletion, *sgfp* and *ctap* epitope taggings. (E–G) Southern results for *Anfus3* [*mpkB*] gene replacements for *sgfp*, *ctap* and deletion. Bands produced by the restriction enzymes are compatible with the theoretical map of the *Anfus3* [*mpkB*] locus. Blue bars indicate the regions where the Southern probes bind.
(TIF)

Table S1 SEQUEST Multiple Consensus Report of AnFus3 [MpkB]::cTAP tag and sGFP identifications after nano-LC-ESI-MS2.
(XLS)

Table S2 SEQUEST Multiple Consensus Report of VeA::cTAP tag identifications in wild type after nano-LC-ESI-MS2.
(XLS)

Table S3 SEQUEST Multiple Consensus Report of VeA::cTAP tag identifications in *mpkB*Δ strain after nano-LC-ESI-MS2.
(XLS)

Table S4 SEQUEST Multiple Consensus Report of AnSte7 [MkkB]::cTAP tag identifications after nano-LC-ESI-MS2.
(XLS)

Table S5 SEQUEST Multiple Consensus Report of AnSte7 [MkkB]::cTAP tag identifications in *steC*Δ strain after nano-LC-ESI-MS2.
(XLS)

Table S6 SEQUEST Multiple Consensus Report of AnSte50 [SteD]::cTAP tag identifications after nano-LC-ESI-MS2.
(XLS)

Table S7 Fungal strains used in this study.
(DOC)

Table S8 Plasmids employed in this study.
(DOC)

Table S9 Oligonucleotides utilized for plasmid constructions and northern hybridizations.
(DOC)

Video S1 Time-lapse analysis of the subcellular movements of the AnSte7-GFP fusion along the fungal cells. Individual focal planes were captured with a spinning disc confocal microscope at 2 min intervals (total 26 min). AnSte7 protein moves in an internuclear manner. The nuclei were visualized by mRFP::Histone2A fusion protein. Green spot leaves the first nucleus and shortly touches the plasma membrane (zigzag movement) and sticks to the envelope of the next nucleus. Some spots of the AnSte7 are static (immobile dot at the hyphal tip). The video is presented at a rate of 5 frames/second.
(MOV)

Video S2 Time-lapse capture of the subcellular movements of the AnSte50-GFP fusion within the fungal cell. Single focal planes were captured at 2 min intervals (total 22 min). AnSte50 protein moves to the nucleus (red), hits the nuclear envelope or nucleus and moves in a retrograde direction. Due to the single focal plane, spot disappears at 6 min. After 6 min, spot movement can be tracked again. The video speed is 5 frames/second.
(MOV)

Video S3 Time-lapse analysis of the subcellular movements of the AnSte7-AnFus3 complexes (yellow dot) along the fungal hypha. AnSte7-Fus3 complexes move in a retro and anterograde direction in comparison to the hyphal tip (upper left). These complexes move between the nuclei, which were visualized by mRFP::Histone2A fusion. Single focal layer images were captured at 1 min intervals (total 17 min). The video was produced at a setting 5 frames/second.
(MOV)

Video S4 Time-lapse analysis of the subcellular dynamics of the AnSte50-AnFus3 complexes (yellow dot) in the fungal hypha. Single focal pictures were taken at 2 min intervals (total 58 min, 48 min is shown). This movie shows the movement of the AnSte50-AnFus3 complexes between two nuclei. Protein complexes (yellow dot) leave the first nucleus (visualized by mRFP::Histone2A fusion) and move to the second one. While moving to the second one, the complexes slightly touch the plasma membrane. Video is presented by using the setting 5 frames/second.
(MOV)

Video S5 Retrograde translocation of the AnSte11-Ste7 protein complexes along the fungal hypha. AnSte11-Ste7 complexes move backwards from the hyphal tip. They leave the membrane and touch the nucleus. They also accumulate at septa (faint immobile yellow dot). Single focal planes were captured at 2 min interval (total 54 min, 28 min is shown). The video is presented at the speed of 5 frames/second.
(MOV)

Video S6 Time-lapse analysis of the migration of AnSte11-Ste7 complexes from the plasma membrane to the nucleus. Membrane-tethered complexes (yellow spots) slowly move to the nucleus (red). The movie was captured at 1 min interval (total 39 min). 5 frames/second.
(MOV)

## References

[1] Marshall CJ (1994). MAP kinase kinase kinase, MAP kinase kinase and MAP kinase.. Curr Opin Genet Dev.

[2] Yu JH (2010). Regulation of development in *Aspergillus nidulans* and *Aspergillus fumigatus*.. Mycobiology.

[3] Roman E, Arana DM, Nombela C, Alonso-Monge R, Pla J (2007). MAP kinase pathways as regulators of fungal virulence.. Trends Microbiol.

[4] Saito H (2010). Regulation of cross-talk in yeast MAPK signaling pathways.. Curr Opin Microbiol.

[5] Bardwell L (2004). A walk-through of the yeast mating pheromone response pathway.. Peptides.

[6] Bayram O, Braus GH (2012). Coordination of secondary metabolism and development in fungi: the velvet family of regulatory proteins.. FEMS Microbiol Rev.

[7] Hao N, Nayak S, Behar M, Shanks RH, Nagiec MJ (2008). Regulation of cell signaling dynamics by the protein kinase-scaffold Ste5.. Mol Cell.

[8] Inouye C, Dhillon N, Thorner J (1997). Ste5 RING-H2 domain: role in Ste4-promoted oligomerization for yeast pheromone signaling.. Science.

[9] Pryciak PM, Huntress FA (1998). Membrane recruitment of the kinase cascade scaffold protein Ste5 by the Gbetagamma complex underlies activation of the yeast pheromone response pathway.. Genes Dev.

[10] Good M, Tang G, Singleton J, Remenyi A, Lim WA (2009). The Ste5 scaffold directs mating signaling by catalytically unlocking the Fus3 MAP kinase for activation.. Cell.

[11] Truckses DM, Bloomekatz JE, Thorner J (2006). The RA domain of Ste50 adaptor protein is required for delivery of Ste11 to the plasma membrane in the filamentous growth signaling pathway of the yeast *Saccharomyces cerevisiae*.. Mol Cell Biol.

[12] Wu C, Jansen G, Zhang J, Thomas DY, Whiteway M (2006). Adaptor protein Ste50p links the Ste11p MEKK to the HOG pathway through plasma membrane association.. Genes Dev.

[13] van Drogen F, Stucke VM, Jorritsma G, Peter M (2001). MAP kinase dynamics in response to pheromones in budding yeast.. Nat Cell Biol.

[14] Good MC, Zalatan JG, Lim WA (2011). Scaffold proteins: hubs for controlling the flow of cellular information.. Science.

[15] Maeder CI, Hink MA, Kinkhabwala A, Mayr R, Bastiaens PI (2007). Spatial regulation of Fus3 MAP kinase activity through a reaction-diffusion mechanism in yeast pheromone signalling.. Nat Cell Biol.

[16] Lev S, Sharon A, Hadar R, Ma H, Horwitz BA (1999). A mitogen-activated protein kinase of the corn leaf pathogen *Cochliobolus heterostrophus* is involved in conidiation, appressorium formation, and pathogenicity: diverse roles for mitogen-activated protein kinase homologs in foliar pathogens.. Proc Natl Acad Sci U S A.

[17] Di Pietro A, Garcia-MacEira FI, Meglecz E, Roncero MI (2001). A MAP kinase of the vascular wilt fungus *Fusarium oxysporum* is essential for root penetration and pathogenesis.. Mol Microbiol.

[18] Zhao X, Kim Y, Park G, Xu JR (2005). A mitogen-activated protein kinase cascade regulating infection-related morphogenesis in *Magnaporthe grisea*.. Plant Cell.

[19] Li D, Bobrowicz P, Wilkinson HH, Ebbole DJ (2005). A mitogen-activated protein kinase pathway essential for mating and contributing to vegetative growth in *Neurospora crassa*.. Genetics.

[20] Chen C, Harel A, Gorovoits R, Yarden O, Dickman MB (2004). MAPK regulation of sclerotial development in *Sclerotinia sclerotiorum* is linked with pH and cAMP sensing.. Mol Plant Microbe Interact.

[21] Park G, Pan S, Borkovich KA (2008). Mitogen-activated protein kinase cascade required for regulation of development and secondary metabolism in *Neurospora crassa*.. Eukaryot Cell.

[22] Idnurm A, Bahn YS, Nielsen K, Lin X, Fraser JA (2005). Deciphering the model pathogenic fungus *Cryptococcus neoformans*.. Nat Rev Microbiol.

[23] Rispail N, Soanes DM, Ant C, Czajkowski R, Grunler A (2009). Comparative genomics of MAP kinase and calcium-calcineurin signalling components in plant and human pathogenic fungi.. Fungal Genet Biol.

[24] Wei H, Requena N, Fischer R (2003). The MAPKK kinase SteC regulates conidiophore morphology and is essential for heterokaryon formation and sexual development in the homothallic fungus *Aspergillus nidulans*.. Mol Microbiol.

[25] Paoletti M, Seymour FA, Alcocer MJ, Kaur N, Calvo AM (2007). Mating type and the genetic basis of self-fertility in the model fungus *Aspergillus nidulans*.. Curr Biol.

[26] Vallim MA, Miller KY, Miller BL (2000). Aspergillus SteA (sterile12-like) is a homeodomain-C2/H2-Zn+2 finger transcription factor required for sexual reproduction.. Mol Microbiol.

[27] Axelrod DE, Gealt M, Pastushok M (1973). Gene control of developmental competence in *Aspergillus nidulans*.. Dev Biol.

[28] Atoui A, Bao D, Kaur N, Grayburn WS, Calvo AM (2008). *Aspergillus nidulans* natural product biosynthesis is regulated by *mpkB*, a putative pheromone response mitogen-activated protein kinase.. Appl Environ Microbiol.

[29] Bok JW, Keller NP (2004). LaeA, a regulator of secondary metabolism in *Aspergillus spp*.. Eukaryot Cell.

[30] Bayram O, Krappmann S, Ni M, Bok JW, Helmstaedt K (2008). VelB/VeA/LaeA complex coordinates light signal with fungal development and secondary metabolism.. Science.

[31] Sarikaya Bayram O, Bayram O, Valerius O, Park HS, Irniger S (2010). LaeA control of velvet family regulatory proteins for light-dependent development and fungal cell-type specificity.. PLoS Genet.

[32] Jun SC, Lee SJ, Park HJ, Kang JY, Leem YE (2011). The MpkB MAP kinase plays a role in post-karyogamy processes as well as in hyphal anastomosis during sexual development in *Aspergillus nidulans*.. J Microbiol.

[33] Ni M, Yu JH (2007). A novel regulator couples sporogenesis and trehalose biogenesis in *Aspergillus nidulans*.. PLoS ONE.

[34] Teague MA, Chaleff DT, Errede B (1986). Nucleotide sequence of the yeast regulatory gene STE7 predicts a protein homologous to protein kinases.. Proc Natl Acad Sci U S A.

[35] Fleissner A, Leeder AC, Roca MG, Read ND, Glass NL (2009). Oscillatory recruitment of signaling proteins to cell tips promotes coordinated behavior during cell fusion.. Proc Natl Acad Sci U S A.

[36] Zheng CF, Guan KL (1993). Cloning and characterization of two distinct human extracellular signal-regulated kinase activator kinases, MEK1 and MEK2.. J Biol Chem.

[37] van Drogen F, Peter M (2001). MAP kinase dynamics in yeast.. Biol Cell.

[38] Hu CD, Chinenov Y, Kerppola TK (2002). Visualization of interactions among bZIP and Rel family proteins in living cells using bimolecular fluorescence complementation.. Mol Cell.

[39] Hoff B, Kuck U (2005). Use of bimolecular fluorescence complementation to demonstrate transcription factor interaction in nuclei of living cells from the filamentous fungus *Acremonium chrysogenum*.. Curr Genet.

[40] Brodhun F, Feussner I (2011). Oxylipins in fungi.. FEBS J.

[41] Nayak T, Szewczyk E, Oakley CE, Osmani A, Ukil L (2006). A versatile and efficient gene-targeting system for *Aspergillus nidulans*.. Genetics.

[42] Busch S, Eckert SE, Krappmann S, Braus GH (2003). The COP9 signalosome is an essential regulator of development in the filamentous fungus *Aspergillus nidulans*.. Mol Microbiol.

[43] Bayram O, Sari F, Braus GH, Irniger S (2009). The protein kinase *ImeB* is required for light-mediated inhibition of sexual development and for mycotoxin production in *Aspergillus nidulans*.. Mol Microbiol.

[44] Punt PJ, van den Hondel CA (1992). Transformation of filamentous fungi based on hygromycin B and phleomycin resistance markers.. Methods Enzymol.

[45] Krappmann S, Jung N, Medic B, Busch S, Prade RA (2006). The *Aspergillus nidulans* F-box protein GrrA links SCF activity to meiosis.. Mol Microbiol.

[46] Sikorski RS, Hieter P (1989). A system of shuttle vectors and yeast host strains designed for efficient manipulation of DNA in *Saccharomyces cerevisiae*.. Genetics.

[47] Bruckner S, Kohler T, Braus GH, Heise B, Bolte M (2004). Differential regulation of Tec1 by Fus3 and Kss1 confers signaling specificity in yeast development.. Curr Genet.

[48] Southern EM (1975). Detection of specific sequences among DNA fragments separated by gel electrophoresis.. J Mol Biol.

[49] Brown T, Mackey K (1997). Analysis of RNA by Northern and slot blot hybridization. Current protocols in molecular biology.

[50] Studier FW (2005). Protein production by auto-induction in high density shaking cultures.. Protein Expr Purif.

[51] Maerz S, Dettmann A, Ziv C, Liu Y, Valerius O (2009). Two NDR kinase-MOB complexes function as distinct modules during septum formation and tip extension in *Neurospora crassa*.. Mol Microbiol.

[52] Brakhage AA, Browne P, Turner G (1994). Analysis of the regulation of penicillin biosynthesis in *Aspergillus nidulans* by targeted disruption of the *acvA* gene.. Mol Gen Genet.

