# Supplementary figures and images for: The Aspergillus nidulans MAPK Module AnSte11-Ste50-Ste7-Fus3 Controls Development and Secondary Metabolism
Source: PLoS Genet. 2012 Jul 19;8(7):e1002816. doi: 10.1371/journal.pgen.1002816 (PMC3400554; doi:10.1371/journal.pgen.1002816)

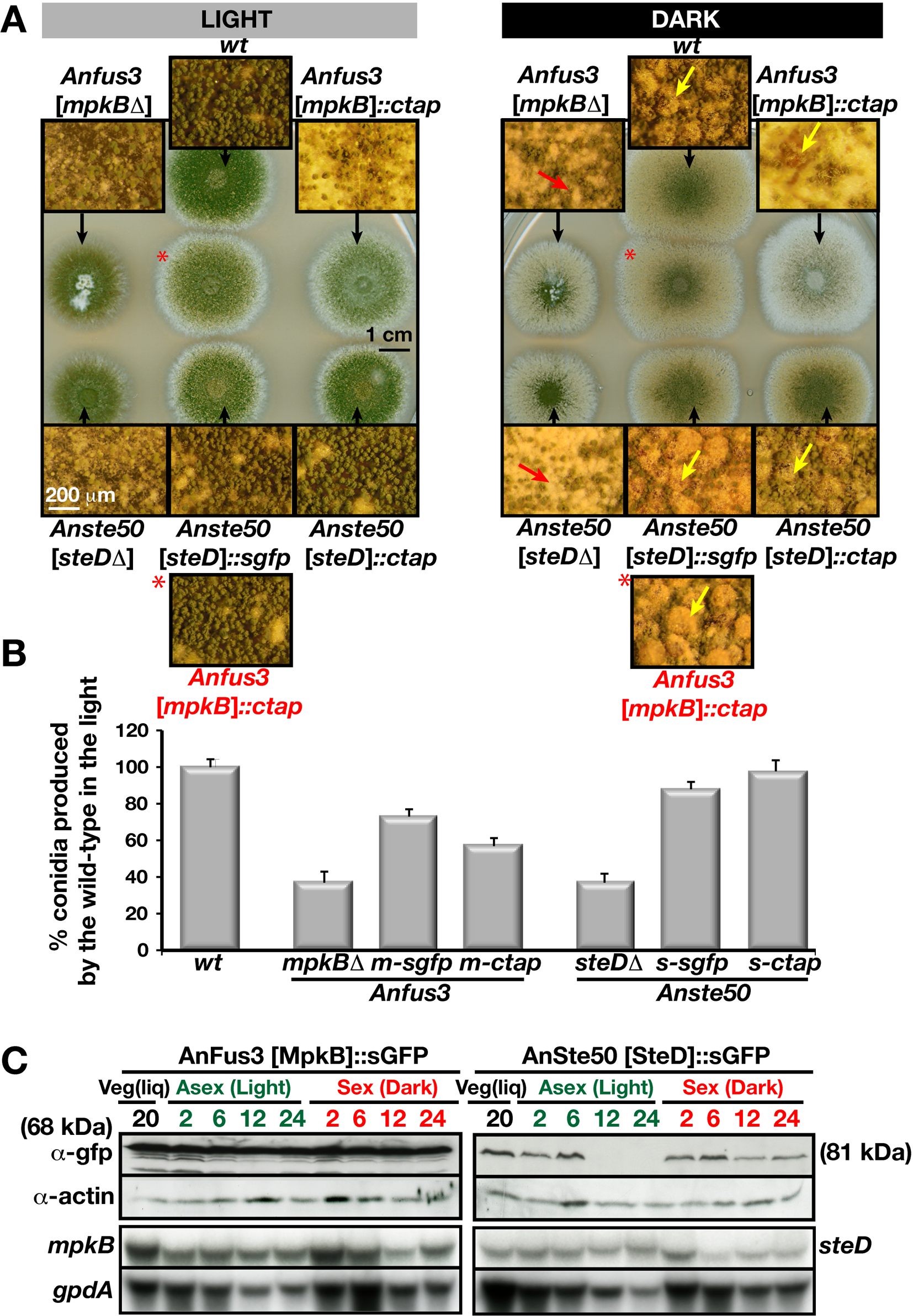

Supplement: Figure S1 — Functionality of AnFus3 [MpkB] and AnSte50 [SteD]::sGFP and TAP fusions for fungal growth, sexual development. (A) Phenotypes of the wild type, Anfus3 [mpkBΔ], Anste50 [steDΔ], Anfus3 [mpkB]::sgfp, Anfus3 [mpkB]::ctap, Anste50 [steD]::sgfp, Anste50 [steD]::ctap strains incubated under dark and light conditions for 5 days at 37°C. Black frames are the stereomicroscopic images of the colonies on the plates. Mature fruiting bodies are indicated by yellow arrows. Anfus3 [mpkBΔ] and Anste50 [steDΔ] strains cannot produce mature cleistotheica (indicated by red arrows) instead form nest-like structures. (B) Quantification of the asexual conidiations of the strains from (A). Reduced asexual sporulation seen in Anfus3 [mpkB] and Anste50 [steD] mutants. Replacement strains sporulate more efficient than the deletion strains. (C) Expression of AnSte50 [SteD] and AnFus3 [MpkB]::sGFP proteins during different developmental stages (vegetative, asexual and sexual, respectively). 68 kDA AnFus3 [MpkB] and 81 kDA AnSte50 [SteD]::sGFP fusion proteins were detected by α-gfp. Constitutive transcript levels of the mpkB and steD genes from the same experiments. Actin levels served as loading control for immunoblotting (80 µg in each lane), and internal gpdA expression was used as control for Northern hybridizations. 20 µg RNA was loadeded in each sample. (TIF) [file pgen.1002816.s001.tif]

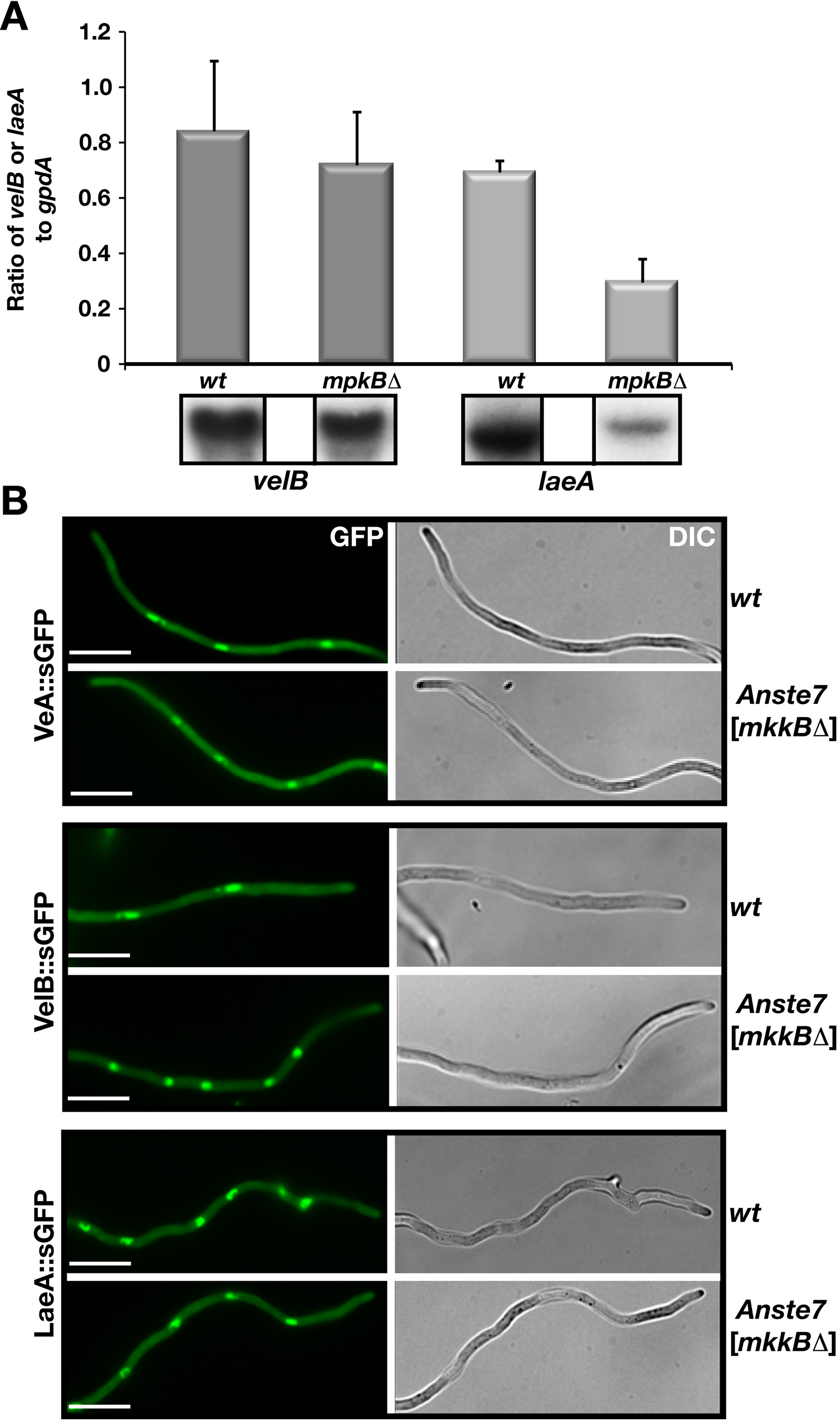

Supplement: Figure S2 — Transcript levels of velB and laeA and cellular localizations of the velvet complex components in the wild type and Anste7 [mkkBΔ] strain. (A) Transcript levels of velB and laeA in the wild type and mpkB mutant background. RNA levels of velB and laeA from two different time points (24 and 72 hours) were quantified and normalized to the internal control gene expression gpdA. velB levels do not change significantly, but laeA transcript is drastically downregulated. Black bars represent standard deviations. (B) Localization patterns of VeA, VelB and LaeA::sGFP fusions in the wild type and mpkB mutant background. Fungal strains were grown in the darkness for 24 hours at 30°C and pictures were taken in a fluorescence microscope. Scale bars represent 10 µm. (TIF) [file pgen.1002816.s002.tif]

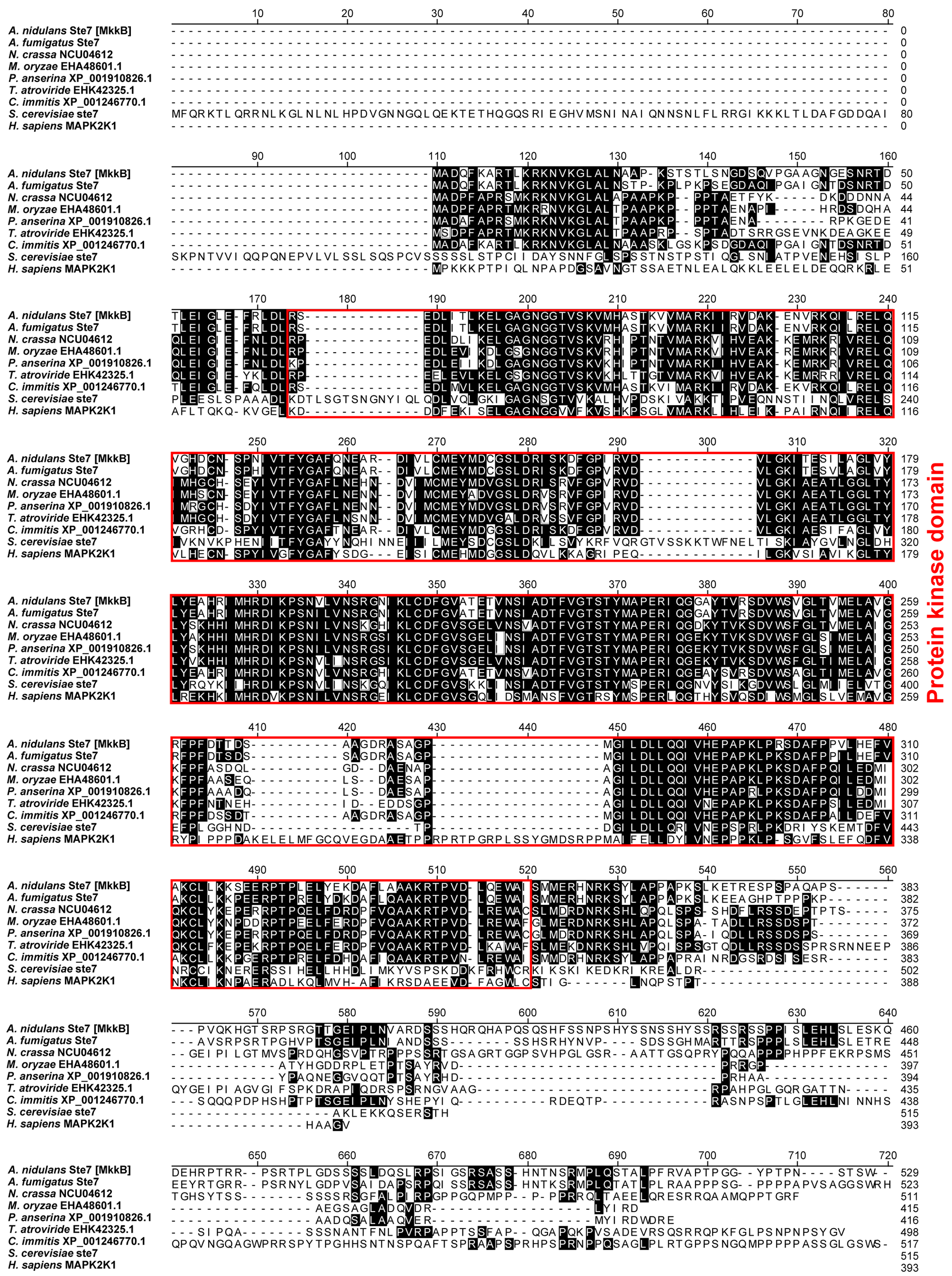

Supplement: Figure S3 — Global alignment of the AnSte7 [MkkB] of Aspergillus nidulans with other eukaryotic MAPK Kinase homologs. A. nidulans AnSte7 [MkkB] (ANID_03422) was aligned with the amino acid sequences from Aspergillus fumigatus Afu3g05900, Neurospora crassa MAPK Kinase (NCU04612), Magnaporthe oryzae (EHA48601.1), Podospora anserina (XP_001910826.1), Trichoderma atroviride (EHK42325.1), Coccidioides immitis (XP_001246770.1), Saccharomyces cerevisiae Ste7p, and Homo sapiens MAPK2K1. Conserved protein kinase domains and the central part showing higher similarity are indicated with red rectangle. N- and C-terminal sequences of the kinase proteins show less similarity. S. cerevisiae and H. sapiens proteins often break the alignment. Filamentous fungus kinases show higher similarity to the AnSte7 protein. (TIF) [file pgen.1002816.s003.tif]

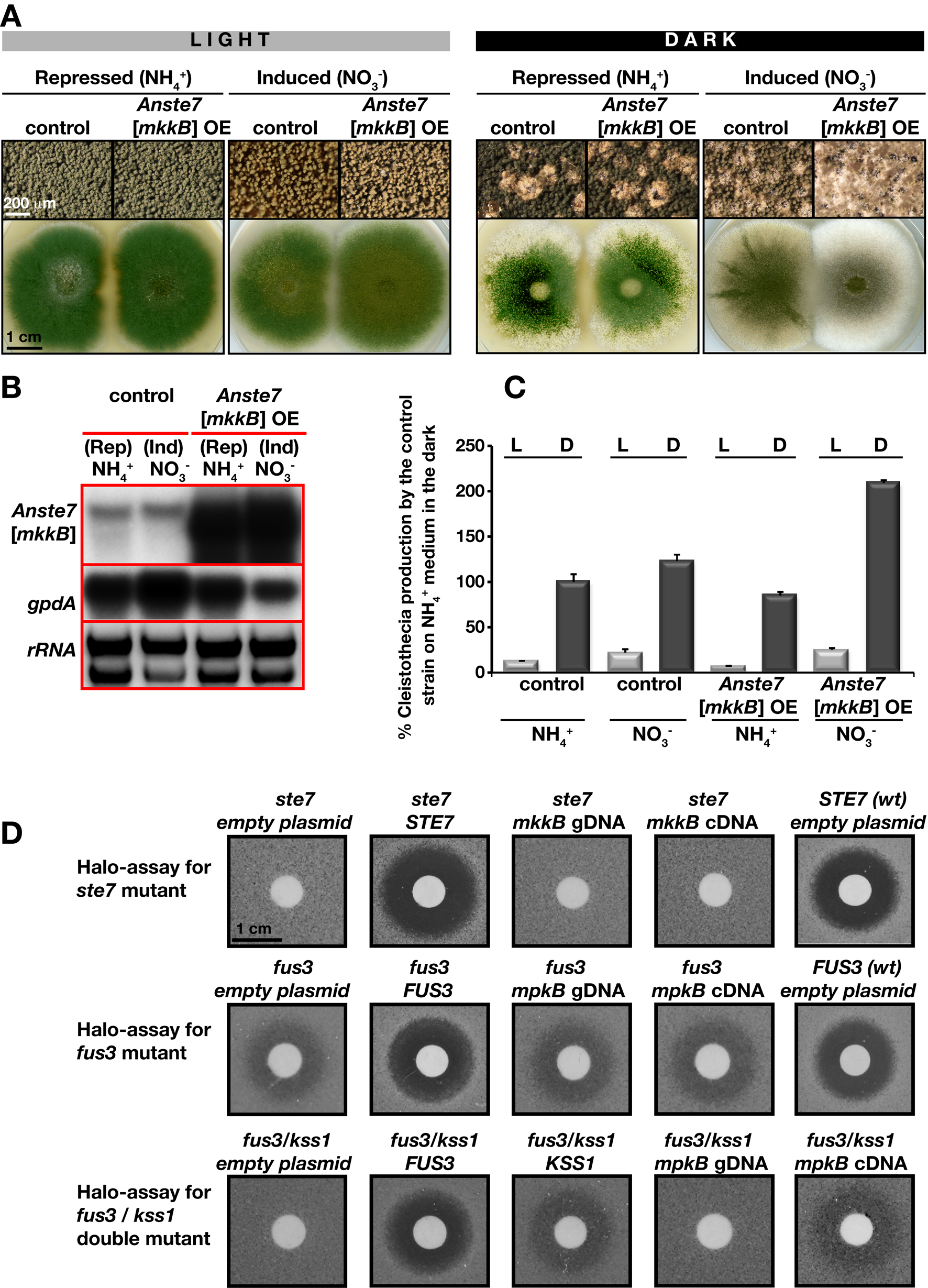

Supplement: Figure S4 — Increased sexual development caused by overexpression of Anste7 [mkkB] gene and functional complementation assays in yeast. (A) Growth of the control (empty plasmid carrying strain) and Anste7 [mkkB] OE (under niiA promoter) strains under white light (90 µWm2) and dark conditions on repressing (NH4 +) and inducing (NO3 −) media. Lower panel shows the plate pictures, upper squares are the stereomicroscopic images taken from the plates. 1×10^4^ spores were point-inoculated and grown at 37°C for 5 days. (B) Validation of Anste7 [mkkB] overexpression by Northern blot. gpdA transcript levels and rRNA were used as equal loading controls. Total 20 µg RNA was applied in each lane. (C) Quantification of cleistothecia production from (A). Increased cleistotheica production in Anste7 [mkkB] OE strain in the dark on nitrate containing inducing medium. Vertical lines are the standard errors originating from different counts. L; light, D; dark. Rep; repressed, Ind; induced. (D) Either cDNA or ORF of Anste7 [mkkB] and Anfus3 [mpkB] expressed under yeast STE7 or FUS3 promoters in a centromeric self-replicating plasmid. These constructs were expressed in the respective fus3, ste7 and fus3/kss1 double mutants. Strains were grown in the presence of 15 µg alpha factor given on the paper discs at 30°C for 3 days. Alpha factor in wild type (empty plasmid) and complementation strains (STE7 in ste7 mutant, FUS3 in fus3 mutant, FUS3 in fus3/kss1 mutant) results in a strong growth inhibition (halo). ste7 and fus3/kss1 mutants do not show any response to the pheromone treatment. fus3 mutant exhibits a reduced response (cloudy halo). AnSte7 and Fus3 do not remediate the halo phenotype of the ste7 and fus3 mutants. mpkB cDNA partially restores the pheromone response of the fus3/kss1 double mutant. (TIF) [file pgen.1002816.s004.tif]

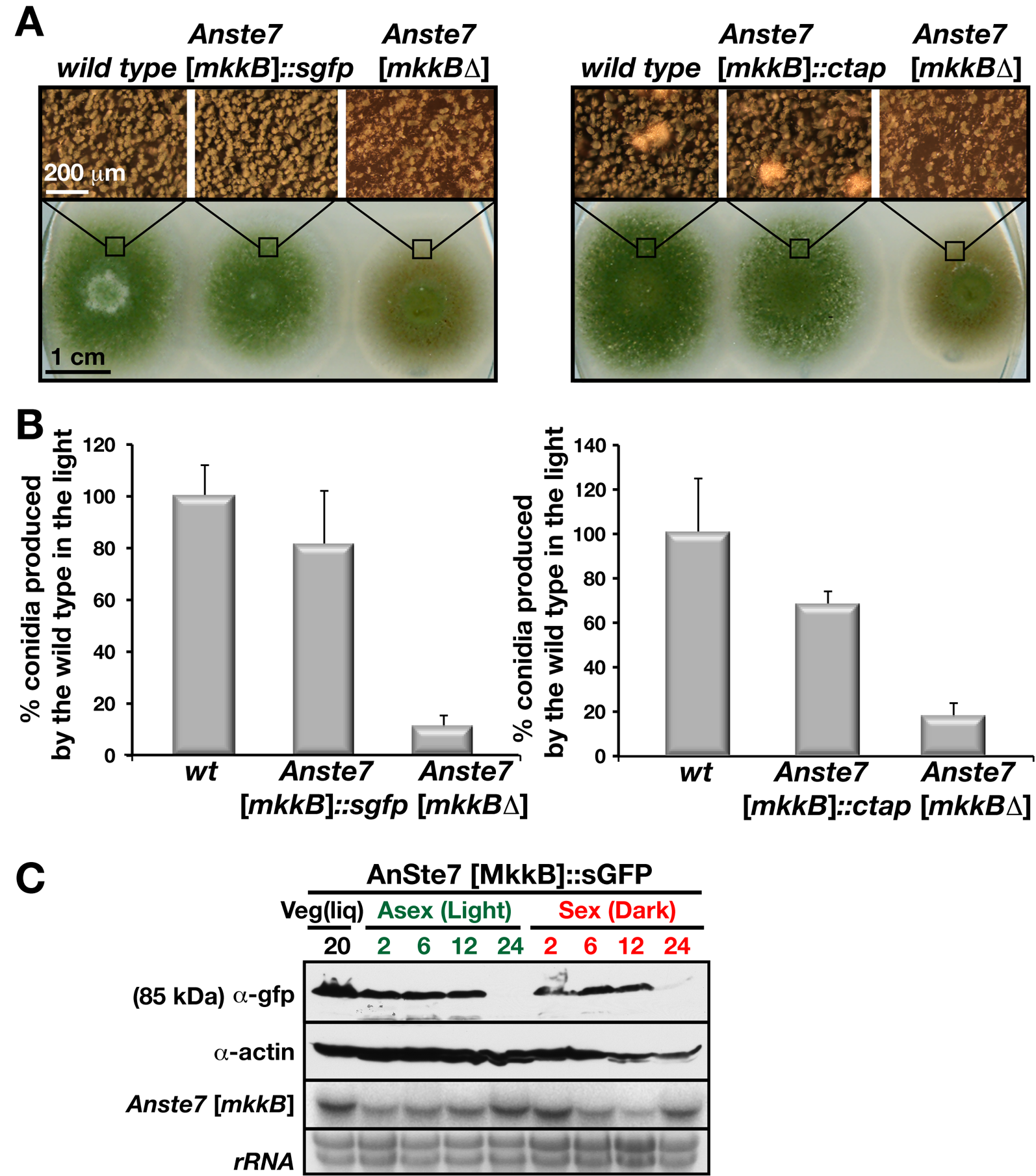

Supplement: Figure S5 — Functionality of the AnSte7 [MkkB]::sGFP and cTAP tag fusions for fungal growth and sexual development. (A) Development of the wild type, Anste7 [mkkB]::sgfp, Anste7 [mkkB]::ctap, Anste7 [mkkBΔ] strains on plates incubated under white light (90 µWm2). Anste7 [mkkB]::sgfp and ctap look like the wild type strain. (B) Conidia production capacities of the strains from (A). Strains carrying Anste7 [mkkB]::sgfp and ctap constructs produce similar levels of conidia of the wild type levels. Vertical bars are the standard deviations of quantifications. Same spore number was used for inoculation as in Figure S4. (C) AnSte7 [MkkB] protein levels under native promoter during different developmental stages of A. nidulans. Strains were grown vegetatively 20 h, and transferred on plates and incubated under light conditions (2, 6, 12, 24 hours) for asexual development and dark for sexual development. Protein undergoes degradation during 24 h asexual and sexual time points. Anste7 [mkkB] transcripts are expressed constitutively during different stages. Actin levels and ethidium bromide stained ribosomal RNA served as loading controls. (TIF) [file pgen.1002816.s005.tif]

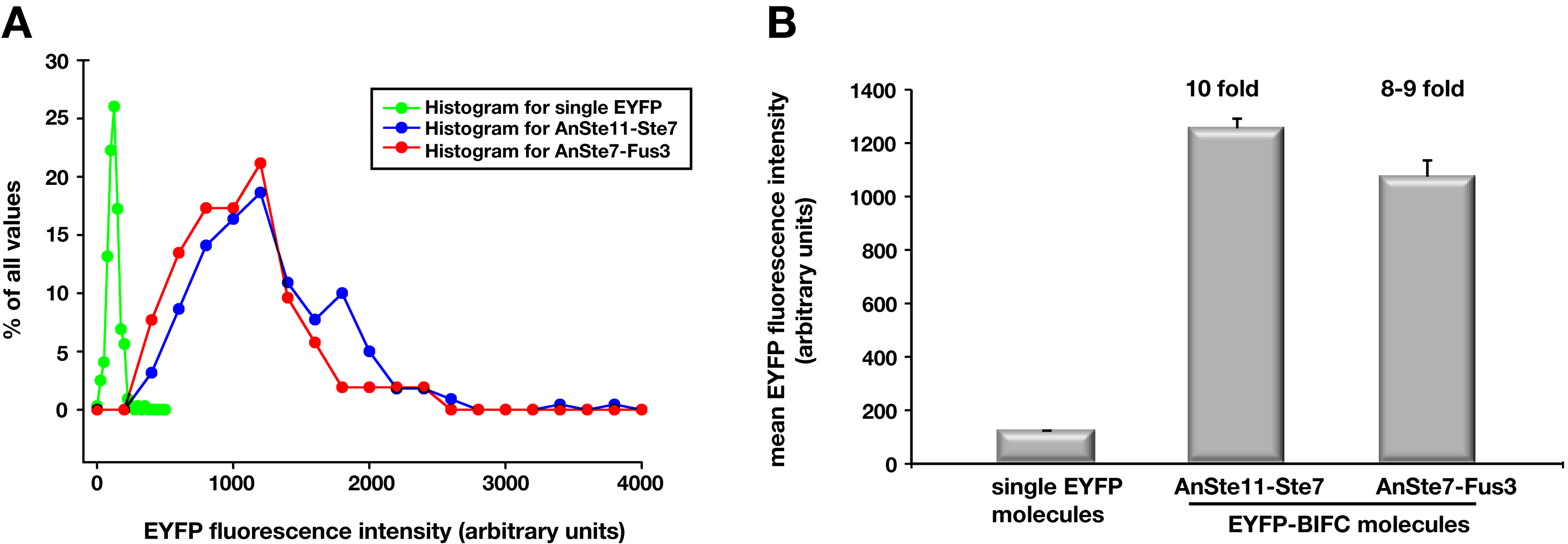

Supplement: Figure S6 — Quantification of the EYFP fluorescence intensities from single EYFP and AnSte11-Ste7 and AnSte7-Fus3 BIFC complexes. (A) The intensity of the spots produced by the AnSte11-Ste7 and AnSte7-Ste3 complexes were measured (see Figure 7 for examples). The intensities are comparable, although the values tend to be higher for AnSte11-Ste7 (blue) than for AnSte7-Fus3 (red). To obtain an estimate of the number of molecules in the complexes, these values were compared to the intensity of single EYFP molecules attached to coverglasses (green). (B) The bar graphs indicate the average values for the intensities (obtained from the same datasets as in A). The intensity of the complexes is ∼9-fold (AnSte7-Fus3) or ∼10-fold (AnSte11-Ste7) higher than that of single EYFP molecules, suggesting the presence of 9 to 10 molecules in a complex. The bars show the mean and standard error; 50–300 spots were analyzed for each condition. (TIF) [file pgen.1002816.s006.tif]

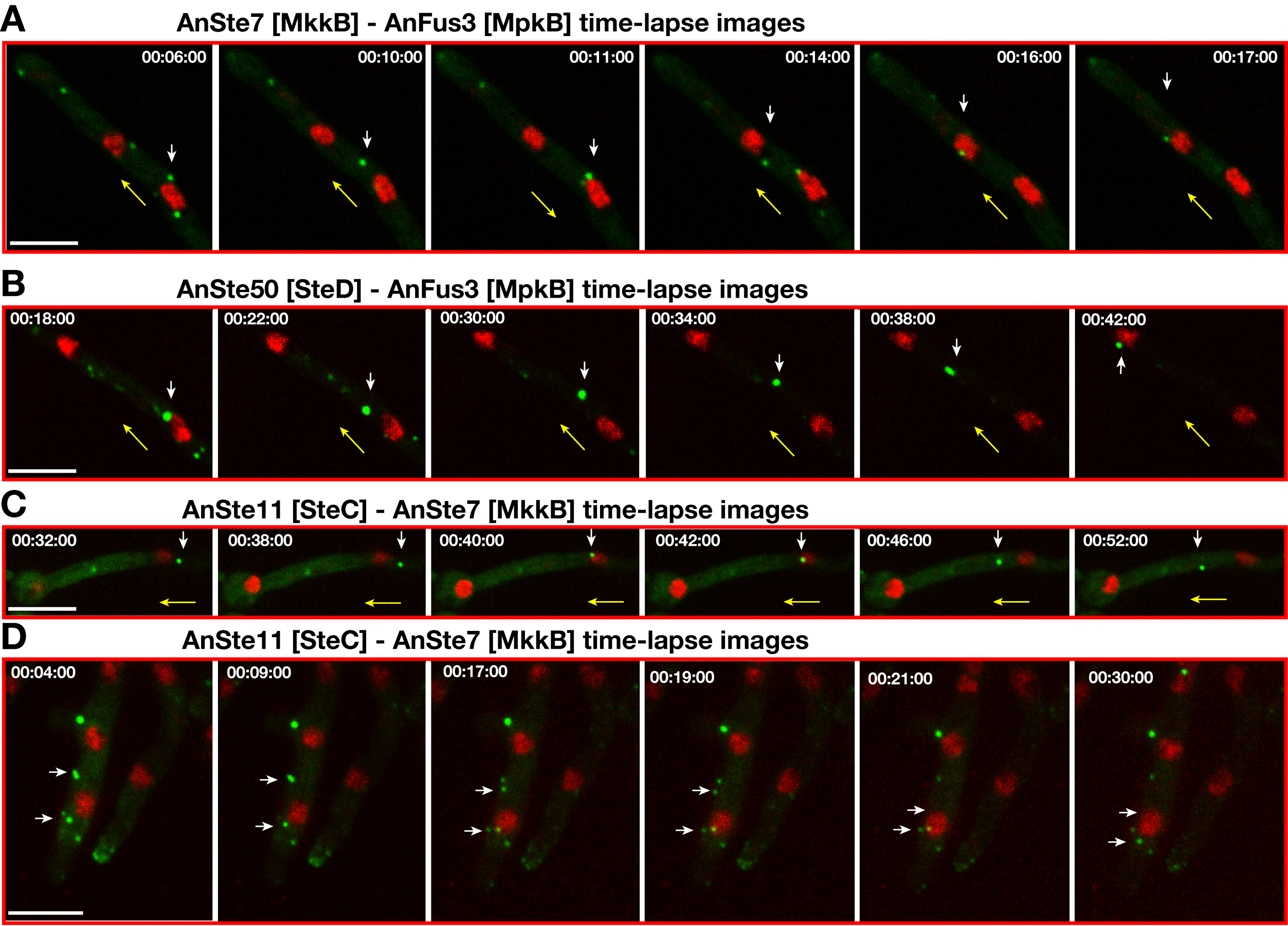

Supplement: Figure S7 — Spatial movements of the AnSte7-AnFus3, AnSte50-AnFus3, AnSte11-AnSte7 binary complexes within the fungal cells. (A) Movement of the AnSte7 [MkkB]-AnFus3 [MpkB] complex (white arrow) that touches the nucleus during intracellular translocations (17 min) (Video S3). Yellow arrows indicate the direction of the movements. (B) A movement of the AnSte50-AnFus3 complexes between two nuclei. Complex leaves the first nucleus, and slightly touches the membrane (a small deviation to upper side) reaches to the second nucleus (Video S4). (C) A horizontal backwards movement of the AnSte11 [SteC]-AnSte7 [MkkB] complexes from hyphal tip. Complexes touch the nucleus during bypass (Video S5). (D) A vertical movement of the AnSte11-AnSte7 complexes from membrane to the nuclear envelope (Video S6). White arrows indicate the YFP spots representing the binary complexes moving to the nucleus. (TIF) [file pgen.1002816.s007.tif]

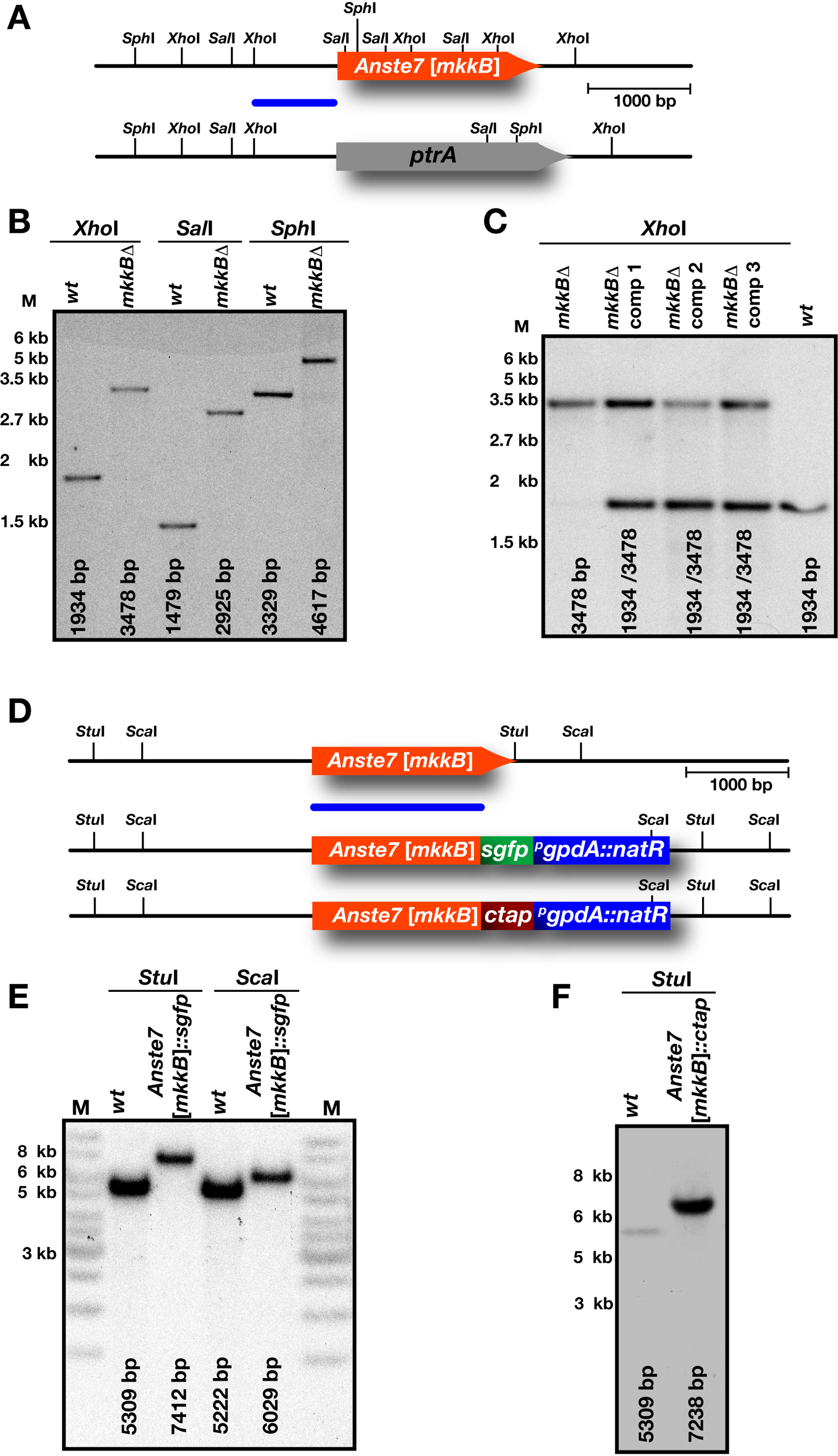

Supplement: Figure S8 — Southern hybridizations for the gene replacement experiments involving Anste7 [mkkB] locus. (A) A comparative depiction of the genomic Anste7 [mkkB] (AN3422) and the deletion of the locus by the selection marker pyrithiamine resistance gene, ptrA. Blue bar represents the Southern probe used in hybridizations. (B–C) Southern hybridization results of the mkkB deletion and complementation strains. Sizes of the restriction bands confirm the gene replacement and ectopic complementation of the knock-out strain by the complementation plasmid. Sizes of the restriction fragments are given in base pairs. (D) Schematic drawings of the Anste7 [mkkB] locus gene replacements by Anste7 [mkkB]::sgfp::natR and Anste7 [mkkB]::ctap::natR. The cutting sites of the common restriction enzymes are indicated in the theoretical maps. (E–F) Southern results of the Anste7 [mkkB]::sgfp::natR and Anste7 [mkkB]::ctap::natR strains in comparison to the wild type locus. Bands released by restriction digests are in agreement with the theoretical maps of the replaced loci. (TIF) [file pgen.1002816.s008.tif]

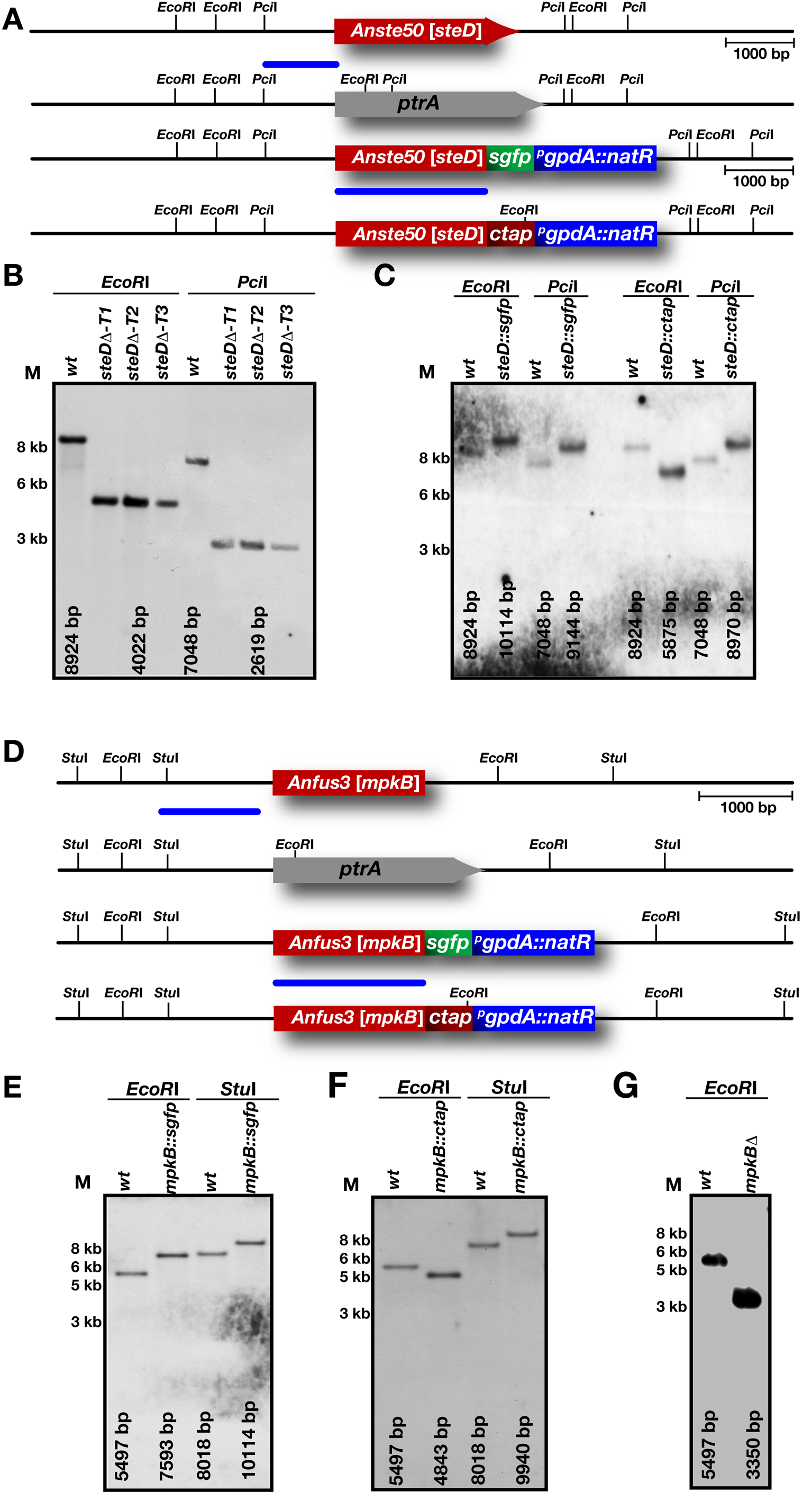

Supplement: Figure S9 — Verification of the gene replacements for Anste50 [steD] and Anfus3 [mpkB] loci. (A) Common restriction enzyme cutting maps of the wild type Anste50 [steD] (AN7252) locus, steDΔ::ptrA, Anste50 [steD]::sgfp::natR, and Anste50 [steD]::ctap::natR gene replacements. Blue lines show the probe binding sites during Southern hybridizations. (B–C) Southern hybridizations of gene replacements in comparison to the wild type Anste50 [steD] locus. Restriction enzymes used during Southern hybridizations are shown at the top of the blot. Lengths of the restriction fragments are given in base pairs. (D) Restriction map of the Anfus3 [mpkB] (AN3719) locus and corresponding gene replacements for deletion, sgfp and ctap epitope taggings. (E–G) Southern results for Anfus3 [mpkB] gene replacements for sgfp, ctap and deletion. Bands produced by the restriction enzymes are compatible with the theoretical map of the Anfus3 [mpkB] locus. Blue bars indicate the regions where the Southern probes bind. (TIF) [file pgen.1002816.s009.tif]
