# Supplementary material for: The Aspergillus nidulans MAPK Module AnSte11-Ste50-Ste7-Fus3 Controls Development and Secondary Metabolism
Source: PLoS Genet. 2012 Jul 19;8(7):e1002816. doi: 10.1371/journal.pgen.1002816 (PMC3400554; doi:10.1371/journal.pgen.1002816)
Supplement: Table S7 — Fungal strains used in this study. (DOC) [file pgen.1002816.s016.doc]

**Table S7. Fungal strains used in this study**

| **Strain** | **Genotype** | **Reference** |
| --- | --- | --- |
| **FGSCA4** | *veA* | FGSC* |
| **FGSCA26** | *veA1*, *biA1* | FGSC* |
| **TNO2A3** | *nkuA***∆**, *pyroA4*, *pyrG89*, *veA1* | [41] |
| **SWH51** | *Anste11* [*steC***∆**]*::argB; pyrG89; argB***∆***::trpC***∆***B; pyroA4; veA1* | [24] |
| **UI89.11** | *Anste12* [*steA***∆**]*::argB, biA1, argB***∆** | [26] |
| **AGB152** | *pyroA4*, *pyrG89*, *veA*+ | [42] |
| **AGB154** | *pabaA1*, *yA2*, *veA*+ | [31] |
| **AGB273** | *^p^veA::veA::ctap* tag, ptrA; pabaA1, yA2; argB*∆*::trpC; trpC801, veA*∆*::argB* | [30] |
| **AGB445** | *nkuA***∆**, *pyroA4*, *pyrG89*, *veA1* **(pME3858)** | [43] |
| **AGB448** | *niiA-niiD*/*pyrG*, *pyroA4*, *pyrG89*, *veA*+ **(pME3160 in AGB152)** | [43] |
| **AGB506** | *^p^gpdA::mrfp::h2A / natR; pyroA4*, *pyrG89*, *veA*+  **(pME3173 in AGB152)** | [31] |
| **AGB551** | *nkuA***∆***::argB*, *pyrG89, pyroA4, veA+* **(TNO2A3 X AGB154)** | This study |
| **AGB552** | *nkuA***∆***::argB, pabaA1*, *yA2*, *veA*+ **(TNO2A3 X AGB154)** | This study |
| **AGB576** | *Anste50* [*steD***∆**]*::ptrA, nkuA***∆***::argB, pabaA1*, *yA2*, *veA*+ | This study |
| **AGB586** | *Anste7* [*mkkB***∆**]*::ptrA*, *nkuA***∆***::argB*, *pyrG89, pyroA4, veA1* | This study |
| **AGB587** | *Anste7* [*mkkB***∆**]*::ptrA*, *pabaA1*, *yA2*, *veA*+ | This study |
| **AGB588** | *mkkB/phleoR, Anste7* [*mkkB***∆**]*::ptrA*, *nkuA***∆***::argB*, *pyrG89, pyroA4, veA1* **(pME3854 in AGB586)** | This study |
| **AGB589** | *mrfp::h2A-pyrG; Anste7* [*mkkB***∆**]*::ptrA*, *nkuA***∆***::argB*, *pyrG89, pyroA4, veA1* **(pME3858 in AGB588)** | This study |
| **AGB590** | *Anste7* [*mkkB*]*::sgfp::natR; nkuA***∆**, *pyroA4*, *pyrG89*, *veA1* **(AnSte7::GFP)** | This study |
| **AGB591** | *mrfp::h2A-pyrG; mkkB::sgfp::natR; nkuA***∆**, *pyroA4*, *pyrG89*, *veA1* **(pME3858 in AGB590)** | This study |
| **AGB592** | *mkkB::sgfp::natR; steC***∆***::argB; pyrG89; argB***∆***::trpC***∆***B; pyroA4; veA1* **(AnSte7::GFP in SWH51)** | This study |
| **AGB593** | *mrfp::h2A-pyrG; mkkB::sgfp::natR; steC***∆***::argB;pyrG89; argB***∆***::trpC***∆***B; pyroA4; veA1* **(pME3858 in AGB592)** | This study |
| **AGB594** | *mrfp::h2A-pyrG; steC***∆***::argB;pyrG89; argB***∆***::trpC***∆***B; pyroA4; veA1* **(pME3858 in SWH51)** | This study |
| **AGB595** | *^p^gpdA::sgfp-phleoR; mkkB***∆**::*ptrA*, *pabaA1*, *yA2*, *veA*+  **(pME3863 in AGB586)** | This study |
| **AGB596** | *^p^gpdA::sgfp-phleoR; pabaA1*, *yA2*, *veA*+  **(pME3863 in AGB154)** | This study |
| **AGB597** | *mkkB::ctap::natR; nkuA***∆**, *pyroA4*, *pyrG89*,*veA*+  **(AnSte7::TAP)** | This study |
| **AGB598** | *mkkB::ctap::natR; steC***∆***::argB;pyrG89; argB***∆***::trpC***∆***B; pyroA4; veA1* **(AnSte7::TAP in SWH51)** | This study |
| **AGB599** | *^P^niiA::n-yfp::Anste11*/*^p^niiD::c-yfp::Anste7-pyrG; ^p^gpdA::mrfp::h2A natR; pyroA4*, *pyrG89*, *veA*+ **(pME3860 in AGB506)** | This study |
| **AGB600** | *^P^niiA::n-yfp::Anste7*/*^p^niiD::c-yfp::Anfus3-pyrG; ^p^gpdA::mrfp::h2A-natR; pyroA4*, *pyrG89*, *veA*+ **(pME3862 in AGB506)** | This study |
| **AGB601** | *^P^niiA::n-yfp::Anfus3*/*^p^niiD::c-yfp::Anste12-pyrG; ^p^gpdA::mrfp::h2A-natR; pyroA4*, *pyrG89*, *veA*+ **(pME3865 in AGB506)** | This study |
| **AGB605** | *^p^niiA::mkkB-pyrG; steC***∆***::argB;pyrG89; argB***∆***::trpC***∆***B; pyroA4; veA1* **(pME3855 in SWH51** | This study |
| **AGB611** | *Anfus3* [*mpkB***∆**]*::ptrA, nkuA***∆***::argB, pabaA1*, *yA2*, *veA*+ | This study |
| **AGB621** | *^P^niiA::n-yfp::Anste7*/*^P^niiD::c-yfp::Anfus3-pyrG; steC***∆***::argB; pyrG89; argB***∆***::trpC***∆***B; pyroA4; veA1* **(pME3862 in SWH51)** | This study |
| **AGB622** | *^P^niiA::n-yfp::Anfus3*/*^P^niiD::c-yfp::laeA-pyrG; ^p^gpdA::mrfp::h2A natR; pyroA4*, *pyrG89*, *veA*+ **(pME3869 in AGB506)** | This study |
| **AGB623** | *^P^niiA::n-yfp::Anfus3*/*^P^niiD::c-yfp::veA-pyrG; ^p^gpdA::mrfp::h2A-natR; pyroA4*, *pyrG89*, *veA*+ **(pME3866 in AGB506)** | This study |
| **AGB624** | *^P^niiA::n-yfp::Anfus3*/*^P^niiD::c-yfp::vosA-pyrG; ^p^gpdA::mrfp::h2A-natR; pyroA4*, *pyrG89*, *veA*+ **(pME3868 in AGB506)** | This study |
| **AGB625** | *^P^niiA::n-yfp::Anfus3*/*^P^niiD::c-yfp::velB-pyrG; ^p^gpdA::mrfp::h2A natR; pyroA4*, *pyrG89*, *veA*+ **(pME3867 in AGB506)** | This study |
| **AGB626** | *^P^niiA::n-yfp::Anste11*/*^P^niiD::c-yfp::Anste50-pyrG; ^p^gpdA::mrfp::h2A-natR; pyroA4*, *pyrG89*, *veA*+ **(pME3870 in AGB506)** | This study |
| **AGB627** | *^P^niiA::n-yfp::Anste7*/*^P^niiD::c-yfp::Anste50-pyrG; ^p^gpdA::mrfp::h2A-natR; pyroA4*, *pyrG89*, *veA*+ **(pME3871 in AGB506)** | This study |
| **AGB629** | *veA::ctap-natR;* *Anfus3* [*mpkB***∆**]*::ptrA; nkuA***∆***::argB, pabaA1*, *yA2* **(pME3711 in AGB611)** | This study |
| **AGB650** | *Anste50* [*steD***∆**]*::ptrA , nkuA***∆***::argB*, *pyrG89, pyroA4, veA+* | This study |
| **AGB651** | *^P^niiA::n-yfp::Anste11*/*^P^niiD::c-yfp::Anste7-pyrG, steD***∆***::ptrA; nkuA***∆***::argB*, *pyrG89, pyroA4, veA+* **(pME3860 in AGB650)** | This study |
| **AGB652** | *^P^niiA::n-yfp::Anste7*/*^p^niiD::c-yfp::Anfus3-pyrG; steD***∆***::ptrA, nkuA***∆***::argB*, *pyrG89, pyroA4, veA+* **(pME3862 in AGB650)** | This study |
| **AGB654** | *Anfus3* [*mpkB*]*::sgfp::natR; nkuA***∆***::argB*, *pyrG89, pyroA4, veA+*  **(AnFus3::GFP)** | This study |
| **AGB655** | *^p^gpdA::mrfp::h2A-pyrG; mpkB::sgfp::natR; nkuA***∆***::argB*, *pyrG89, pyroA4, veA+* **(pME3858 in AGB654)** | This study |
| **AGB656** | *Anfus3* [*mpkB*]*::ctap::natR; nkuA***∆***::argB*, *pyrG89, pyroA4, veA+*  **(AnFus3::TAP)** | This study |
| **AGB657** | *Anste50* [*steD*]*::sgfp::natR; nkuA***∆***::argB*, *pyrG89, pyroA4, veA+*  **(AnSte50::GFP)** | This study |
| **AGB658** | *^p^gpdA::mrfp::h2A-pyrG; steD::sgfp::natR; nkuA***∆**::*argB*, *pyrG89, pyroA4, veA+* **(pME3858 in AGB657)** | This study |
| **AGB659** | *steD::ctap::natR; nkuA***∆***::argB*, *pyrG89, pyroA4, veA+*  **(AnSte50::TAP)** | This study |
| **AGB662** | *^p^niiA:: Anste7* [*mkkB*]*-pyrG; pyroA4*, *pyrG89*, *veA*+  **(pME3855 in AGB152, AnSte7 Overexpression)** | This study |
| **AGB663** | *^P^niiA::n-yfp::Anfus3*/*^p^niiD::c-yfp::Anste50-pyrG; ^p^gpdA::mrfp::h2A-natR; pyroA4*, *pyrG89*, *veA*+  **(pME3927 in AGB506)** | This study |
| **AGB685** | *^P^niiD::e-yfp-pyrG; pyroA4*, *pyrG89*, *veA*+  **(pME3965 in AGB152)** | This study |
| **AGB686** | *^p^gpdA::mpkB::mrfp::h2A^t^-pyrG; Anste7* [*mkkB*]*::sgfp::natR; nkuA***∆**, *pyroA4*, *pyrG89*, *veA1*  **(pME3966 in AGB590)** | This study |
| **AGB687** | *^p^gpdA::mpkB::mrfp::h2A^t^-pyrG; Anste50* [*steD*]*::sgfp::natR; nkuA***∆***::argB*, *pyrG89, pyroA4, veA+*  **(pME3966 in AGB657)** | This study |
| **RH2951** | *MAT****a***  *ura3 his3* | [47] |
| **RH2958** | *MAT****a*** *fus3***∆***::TRP1 ura3 his3 trp1* | [47] |
| **RH3160** | *MAT****a*** *ste7***∆***::kanR ura3 his3* | [47] |
| **RH3165** | *MAT****a*** *fus3***∆***::TRP1 kss1***∆***::kanR ura3 his3 trp1* | [47] |

FGSC* Fungal Genetics Stock Center, Kansas
