# Supplementary material for: The Aspergillus nidulans MAPK Module AnSte11-Ste50-Ste7-Fus3 Controls Development and Secondary Metabolism
Source: PLoS Genet. 2012 Jul 19;8(7):e1002816. doi: 10.1371/journal.pgen.1002816 (PMC3400554; doi:10.1371/journal.pgen.1002816)
Supplement: Table S8 — Plasmids employed in this study. (DOC) [file pgen.1002816.s017.doc]

Table S8. Plasmids employed in this study

| **Plasmid** | **Description** | **Reference** |
| --- | --- | --- |
| **pBluescript II SK** | cloning plasmid | Stratagene |
| **pPTRII** | pyrithiamine resistance (*ptrA*) plasmid | Takara |
| **pRS316** | yeast centromeric plasmid | [46] |
| **pAN8-1** | phleomycin resistance cloning plasmid | [44] |
| **pME3160** | *niiA*/*niiD* expression module with *pyrG* marker | [30] |
| **pME3173** | mRFP::Histone2A plasmid with *natR* marker | [30] |
| **pME3711** | *^p^veA::veA::ctap*/*natR* | [31] |
| **pME3718** | *niiA*/*niiD* expression module with *phleoR* marker | [31] |
| **pME3163** | *velB::sgfp* plasmid with *pyrG* marker | [30] |
| **pME3167** | *veA::sgfp* plasmid with *natR* marker | [30] |
| **pME3190** | *laeA::sgfp* plasmid with *natR* marker | [30] |
| **pME3854** | *Anste7* [*mkkB*] genomic locus in *Stu*I site of pAN8-1 | This study |
| **pME3855** | *^p^niiA:: Anste7* [*mkkB*] *mkkB* cDNA in *Pme*I site of pME3160 | This study |
| **pME3856** | *gpdA* promoter with *Pme*I site for cloning in *Stu*I site of pAN8-1 | This study |
| **pME3857** | mRFP::Histone2A with *phloeR* marker | This study |
| **pME3858** | mRFP::Histone2A with *pyrG* marker | This study |
| **pME3859** | *^P^niiA::n-yfp::Anste11* [*steC*] in *Pme*I site of pME3160 | This study |
| **pME3860** | *^p^niiD::c-yfp::Anste7* [*mkkB*] in *Swa*I site of pME3859 | This study |
| **pME3861** | *^p^niiA::n-yfp::Anste7* [*mkkB*] in *Pme*I site of pME3160 | This study |
| **pME3862** | *^p^niiD::c-yfp::Anfus3* [*mpkB*] in *Swa*I site of pME3861 | This study |
| **pME3863** | *sgfp* under *gpdA* promoter in *Pme*I site of pME3856 | This study |
| **pME3864** | *^p^niiA::n-yfp::Anfus3* [*mpkB*] in *Pme*I site of pME3160 | This study |
| **pME3865** | *^p^niiD::c-yfp::Anste12* [*steA*] in *Swa*I site of pME3864 | This study |
| **pME3866** | *^p^niiD::c-yfp::veA* in *Swa*I site of pME3864 | This study |
| **pME3867** | *^p^niiD::c-yfp::velB* in *Swa*I site of pME3864 | This study |
| **pME3868** | *^p^niiD::c-yfp::vosA* in *Swa*I site of pME3864 | This study |
| **pME3869** | *^p^niiD::c-yfp::laeA* in *Swa*I site of pME3864 | This study |
| **pME3870** | *^p^niiD::c-yfp::Anste50* [*steD*] in *Swa*I site of pME3859 | This study |
| **pME3871** | *^p^niiD::c-yfp::Anste50* [*steD*] in *Swa*I site of pME3861 | This study |
| **pME3927** | *^p^niiD::c-yfp::Anste50* [*steD*] in *Swa*I site of pME3864 | This study |
| **pME3958** | *^p^STE7::mkkB* gDNA*::STE7^t^* | This study |
| **pME3959** | *^p^STE7::mkkB* cDNA*::STE7^t^* | This study |
| **pME3960** | *^p^FUS3::mpkB* gDNA*::FUS3^t^* | This study |
| **pME3961** | *^p^FUS3::mpkB* cDNA*::FUS3^t^* | This study |
| **pME3962** | *5kb STE7* genomic locus in *Sma*I site of pRS316 | This study |
| **pME3963** | *5kb FUS3* genomic locus in *Sma*I site of pRS316 | This study |
| **pME3964** | *5kb KSS1* genomic locus in *Sma*I site of pRS316 | This study |
| **pME3965** | *^p^niiD::e-yfp* in *Swa*I site of pME3160 | This study |
| **pME3966** | *^p^gpdA::mpkB::mrfp::Histone2A^t^* | This study |
