# Supplementary material for: The Aspergillus nidulans MAPK Module AnSte11-Ste50-Ste7-Fus3 Controls Development and Secondary Metabolism
Source: PLoS Genet. 2012 Jul 19;8(7):e1002816. doi: 10.1371/journal.pgen.1002816 (PMC3400554; doi:10.1371/journal.pgen.1002816)
Supplement: Table S9 — Oligonucleotides utilized for plasmid constructions and northern hybridizations. (DOC) [file pgen.1002816.s018.doc]

Table S9. Oligonucleotides utilized for plasmid constructions and northern hybridizations

| **Designation** | **Sequence in 5->3 direction** | **Features** |
| --- | --- | --- |
| 3422-A | CTC GGG CGC TCA TCG TGT GTT G | *mkkB* deletion A |
| 3422-B | CTT GCA ATG GGA CAA GCG ACG | *mkkB* del-nest B |
| 3422-C | CTT TTA CAT TTC GTT ACC AAT GGG ATC CCG TAA TCA ATT GGC GAC GGC GAC TGA AGA TTG | *mkkB* deletion C |
| 3422-D | CAA GAA AGA CAG TAT AAT ACA AAC AAA GAT GCA AGA CCT CTA AAC TAT TCA TGG GCC CC | *mkkB* deletion D |
| 3422-E | CCA CTA GCC GAT GAA CGA GTA TTC | *mkkB* del-nest E |
| 3422-F | GAG CCT CTG TTG TAG TGG GTA GAG | *mkkB* deletion F |
| Comp A | TTT CCC AGA TCT CAT CAT CCG AGA ATA CAT CTT CTT CG | Comp 5 |
| Comp B | TTT CCC AGA TCT CTC TGT TGT AGT GGG TAG AGT ACT C | Comp 3 |
| OZG29 | CTA CTT GTA CAG TTC GTC CAT GCC GTG | *sgfp* stop |
| OZG73 | ATG GTG AGC AAG GGC GAG GAG | *n-yfp* start |
| OZG75 | ATG GCC GAC AAG CAG AAG AAC | *c-yfp* start |
| OZG302 | ATG GCC GAT CAA TTC AAA GCG CG | *mkkB*-ORF 5 |
| OZG303 | TTA GAG GGC CCC CAT ATG GTC GC | *mkkB*-ORF 3 |
| OZG314 | GCA GGC GCT CTA CAT GAG CAT GCC CTG CCC CTG ACC TCT AAA CTA TTC ATG GGC CCC | *mkkB-natR*  fusioner |
| OZG380 | GCC CTT GCT CAC CAT ACC ACC GCT ACC ACC GAG GGC CCC CAT ATG GTC GCC GC | *mkkB-sgfp* fusioner |
| OZG382 | CTT TTT CCA TCT TCT CTT ACC ACC GCT ACC ACC GAG GGC CCC CAT ATG GTC GCC GC | *mkkB-ctap* fusioner |
| OZG385 | GAT CTT TGC CCG GTG TAT GAA ACC | mRFP 5 |
| OZG386 | ATC TGG AGG GGA CAG GCA GTT TAT | mRFP 3 |
| OZG387 | CGT GGC GAT GGA GCG CAT GAT ATA | *n-yfp* 3 |
| OZG388 | GTG GTT CAT GAC CTT CTG TTT CAG GTC | *c-yfp* 3 |
| OZG389 | CGT CTA TAT CAT GCG CTC CAT CGC CAC GAT GGC CGA TCA ATT CAA AGC GCG | *mkkB::n-yfp* 5  fusioner |
| OZG390 | GAA CGA CCT GAA ACA GAA GGT CATG AAC CAC ATG GCC GAT CAA TTC AAA GCG CG | *mkkB::c-yfp* 5  fusioner |
| OZG392 | CTC AGG TAA GTG TTG TAG CAA GGA AG | *steC* stop |
| OZG393 | CAA CGT CTA TAT CAT GCG CTC CAT CGC CAC GAT GCT CAC CTC CAA AGC ATA CGC G | *steC::n-yfp*  fusioner |
| OZG394 | GAA CGA CCT GAA ACA GAA GGT CAT GAA CCA CAT GCT CAC CTC CAA AGC ATA CGC | *steC::c-yfp*  fusioner |
| OZG399 | TTC TAT ATT TGC TGT TGC AGG AGT TG | *steA* stop 3 |
| OZG401 | GAA CGA CCT GAA ACA GAA GGT CAT GAA CCA CAT GTA CTC TCA GCA CGG TGC CCC | *steA::c-yfp*  fusioner |
| OZG402 | ATG CCG CCT CAG GGA GGC TCA AG | *mpkB* start 5 |
| OZG403 | TTT TAG TTG ATT GTC GCA TAA CTT ATG | *mpkB* stop 3 |
| OZG404 | CGT CTA TAT CAT GCG CTC CAT CGC CAC GAT GCC GCC TCA GGG AGG CTC AAG | *mpkB n-yfp*  fusioner |
| OZG405 | GAA CGA CCT GAA ACA GAA GGT CAT GAA CCA CAT GCC GCC TCA GGG AGG CTC AAG | *mpkB c-yfp*  fusioner |
| OZG443 | CCC AGA AGT CCC AGG CCA GTT C | *mpkB* deletion A |
| OZG444 | CAA GAG ATC ATT CTT GAG GCA AAA G | *mpkB* del-nest B |
| OZG445 | CAT TTC GTT ACC AAT GGG ATC CCG TAA TCA ATT CTG CTG CAC CAT GTT GAC TGG | *mpkB* deletion C |
| OZG446 | GAC AGT ATA ATA CAA ACA AAG ATG CAA GAT GTC ATC ACA GTT CTG ATT TAC GAG | *mpkB* deletion D |
| OZG447 | GCT GAC GGC AAT ATA GAA TCA TAC | *mpkB* del-nest E |
| OZG448 | CGA GGC GTT TGG GGA GAC GCT GAG | *mpkB* deletion F |
| OZG470 | GAC CAT CCA GAG GCG GTA ACG | *steD* deletion A |
| OZG471 | GTC GAA GAA TTT GCA TAT CGA TTA TC | *steD* del-nest B |
| OZG472 | CAT TTC GTT ACC AAT GGG ATC CCG TAA TCA ATT GAC GAG AGC GAG CTG ACG AC | *steD* deletion C |
| OZG473 | GAC AGT ATA ATA CAA ACA AAG ATG CAA GAA ACC ATC GCA GGG GCA TAT GC | *steD* deletion D |
| OZG474 | CGC GTG ATC TTT CAC GTA ACC G | *steD* del-nest E |
| OZG475 | CTC CGT AGG TGG AAT CCA AAC AG | *steD* deletion F |
| OZG499 | GCC ACA ACG TCT ATA TCA TGC GCT CCA TCG CCA CGA TGT CCC TCC ATA CCT CCT ACC | *steD n-yfp* start |
| OZG500 | TTA TAA AAC TCC GCC GGG AAG G | *steD* orf stop |
| OZG501 | CGA ACG ACC TGA AAC AGA AGG TCA TGA ACC ACA TGT CCC TCC ATA CCT CCT ACC | *steD c-yfp* start |
| OZG560 | GCC CTT GCT CAC CAT ACC ACC GCT ACC ACC CCG CAT GAT CTC CTC GTA AAT CAG | *mpkB::sGFP fusioner* |
| OZG561 | CTT TTT CCA TCT TCT CTT ACC ACC GCT ACC ACC CCG CAT GAT CTC CTC GTA AAT CAG | *mpkB::TAP fusioner* |
| OZG562 | GCG CTC TAC ATG AGC ATG CCC TGC CCC TGA AGA ATC AAG TGT CGA ATC TTG GAG TTG | *mpkB::natR fusioner* |
| OZG564 | GCC CTT GCT CAC CAT ACC ACC GCT ACC ACC TAA AAC TCC GCC GGG AAG GTT G | *steD::sGFP fusioner* |
| OZG565 | CTT TTT CCA TCT TCT CTT ACC ACC GCT ACC ACC TAA AAC TCC GCC GGG AAG GTT G | *steD::TAP fusioner* |
| OZG566 | GCG CTC TAC ATG AGC ATG CCC TGC CCC TGA AGG CAT GCG ACT TGG ATG AAG C | *steD::natR fusioner* |
| OZG679 | CCA TGA AAT GTT CCT GCT GTG | *Sc STE7 prom 5* |
| OZG680 | GAC CAA CAA CCA ATA TAC CAC G | *Sc STE7 prom 3* |
| OZG681 | ATA TTG GTT GTT GGT CAT GGC CGA TCA ATT CAA AGC G | *mkkB ORF 5* |
| OZG682 | AAT GTT ATC GCA TGC ATT AGA GGG CCC CCA TAT GGT CG | *mkkB ORF 3* |
| OZG683 | TGC ATG CGA TAA CAT TAG TTA GGA AC | *Sc STE7 term 5* |
| OZG684 | GTG ACC ACT CTG GCT TTT TAA CAA G | *Sc STE7 term 3* |
| OZG685 | GAG GCA ATC ATC GTT CCT TTC | *Sc FUS3 prom 5* |
| OZG686 | GAC TGG TTC CAA ACT GCC TTG TAG TTC AAA CGA ACT AGC | *Sc FUS3 prom 3* |
| OZG687 | CAG TTT GGA ACC AGT CAA CAT G | *mpkB ORF 5* |
| OZG688 | GAT TCG ACA CTT GAT TCT CTA CC | *mpkB ORF 3* |
| OZG689 | AAT CAA GTG TCG AAT CCA ACC CGA AGA ACA ATG TAT ACA | *Sc FUS3 term 5* |
| OZG690 | GCA CAC TTG TTG ATG ATT TCA C | *Sc FUS3 term 3* |
| OZG691 | CAG CGA ATA ATT GGG AAA GTT TCA | *Sc KSS1 locus 5* |
| OZG692 | GTT GGA TTA TAC TGC CCA TTA GA | *Sc KSS1 locus 3* |
| OZG733 | TAT GGT GAG CAA GGG CGA GGA G | *e-yfp 5* |
| OZG734 | ATT TTT ACT TGT ACA GCT CGT CCA TGC | *e-yfp 3* |
| OZG735 | GGC GGC TCT GAG GTG CAG TG | *gpdA 5* |
| OZG736 | TTG ACT GGT TCC AAA CGG TGA TGT CTG CTC AAG CGG | *gpdA 3 fusioner* |
| OZG737 | GTT TGG AAC CAG TCA ACA TGG TG | *mpkB start* |
| OZG738 | CGC CTG CAC CAG CTC CCC GCA TGA TCT CCT CGT AAA TCA G | *mpkB mRFP fusioner* |
| OZG739 | GGA GCT GGT GCA GGC GCT GGA GCC | *mRFP 5* |
| OZG740 | CTA AGT ATC CGA GCA AAG GAC TAA C | *Histone2A term 3* |
| *veA* 5 | ATG GAT GGC TAC ACT TGC AGC ACC ACC A | *veA* northern |
| *veA* 3 | TTA ACG CAT GGT GGC AGG CTT TGA GA | *veA* northern |
| *mkkB* 5 | ATG GCC GAT CAA TTC AAA GCG CG | *mkkB* northern |
| *mkkB* 3 | TTA GAG GGC CCC CAT ATG GTC GC | *mkkB* northern |
| *steD* 5 | ATG TCC CTC CAT ACC TCC TAC C | *steD* northern |
| *steD* 3 | TTA TAA AAC TCC GCC GGG AAG G | *steD* northern |
| *mpkB* 5 | ATG CCG CCT CAG GGA GGC TCA AG | *mpkB* northern |
| *mpkB* 3 | TAG TTG ATT GTC GCA TAA CTT ATG | *mpkB* northern |
| *steA* 5 | TTA TGT ACT CTC AGC ACG GTG CCC C | *steA* northern |
| *steA* 3 | TTC TAT ATT TGC TGT TGC AGG AGT TG | *steA* northern |
| *laeA* 5 | GAA TTC ATG TTT GAG ATG GGC CCG GTG GG | *laeA* northern |
| *laeA* 3 | CTC GAG TTA TCT TAA TGG TTT CCT AGC CTG GT | *laeA* northern |
| *aflR* 5 | ATG GAG CCC CCA GCG ATC AGC CAG | *aflR* northern |
| *aflR* 3 | TCA GGC GTG GCG GAG GAT GCT GAT C | *aflR* northern |
| *stcU* 5 | ATG TCC TCC TCC GAT AAT TAC CG | *stcU* northern |
| *stcU* 3 | TTA TCTA AAG GCC CCC CCA TCA ACG | *stcU* northern |
| *tdiA* 5 | ATG GCA CCA AGC AAG ACC GAG | *tdiA* northern |
| *tdiA* 3 | CTA CAG GCC CCG CTC CCT CAG | *tdiA* northern |
| *tdiB* 5 | ATG GCT ACA GAA TAC TGG TCC C | *tdiB* northern |
| *tdiB* 3 | CTA GTT CCT GAA ACC GAA ATC TCC | *tdiB* northern |
| *gpdA* 5 | ATG GCA CCA ACA AAG AAA CAC CAG | *gpdA* northern |
| *gpdA* 3 | CTA TTG GGC ATC AAC CTT GGA G | *gpdA* northern |
